# Overcoming Clusterin-Induced Chemoresistance in Cancer: A Computational Study Using a Fragment-Based Drug Discovery Approach

**DOI:** 10.3390/biology14060639

**Published:** 2025-05-30

**Authors:** Engelo John Gabriel V. Caro, Marineil C. Gomez, Po-Wei Tsai, Lemmuel L. Tayo

**Affiliations:** 1 School of Chemical, Biological, and Materials Engineering and Sciences, Mapúa University, Manila 1002, Philippines; ejgvcaro@mymail.mapua.edu.ph (E.J.G.V.C.); mcgomez@mapua.edu.ph (M.C.G.); 2 School of Graduate Studies, Mapúa University, Manila 1002, Philippines; 3 Department of Food Science, National Taiwan Ocean University, Keelung 202, Taiwan; powei@mail.ntou.edu.tw; 4 Department of Biology, School of Health Sciences, Mapúa University, Makati 1200, Philippines

**Keywords:** clusterin, chemoresistance, cancer biology, fragment-based drug discovery, molecular dynamics

## Abstract

Clusterin is one of the many known proteins implicated in cancer chemoresistance, which hinders the effectiveness of chemotherapy. This study aimed to design novel inhibitors targeting clusterin using fragment-based drug discovery (FBDD). This approach aims to develop new medicines by identifying small, simple molecules known as “fragments” that can bind to a specific target, such as a disease-causing protein. In this study, a primary ligand-binding site and an allosteric site on the clusterin molecule were identified through hotspot analysis. We screened commercially available fragment libraries for anti-cancer activity and applied the “rule of three” to ensure drug-like properties. The highest-affinity fragment underwent “fragment-growing” to develop potential drug candidates. After docking and toxicity screening, 194 candidate drugs were identified. Quantitative structure-activity relationship (QSAR) analysis revealed that the chemical size and complexity of the fragments significantly contributed to their binding affinity. Pharmacokinetic analyses of candidate drugs from FBDD followed by molecular dynamics simulation of the top 1 final candidate drug precursor demonstrated comparatively better affinity (average = −34.01 kcal/mol) than the reference compound (average = −6.15 kcal/mol) and significant ligand flexibility. This study offers a potential strategy to identify fragments or molecules that may serve as drugs against clusterin-related chemoresistance.

## Simple Summary

Clusterin is a protein implicated in cancer chemoresistance, a significant obstacle in effective chemotherapy. This study aimed to design novel inhibitors targeting clusterin using fragment-based drug discovery, which is a method that identifies small, simple molecules, known as “fragments”, which can bind to specific targets, such as disease-causing proteins. In this research, we identified a primary ligand-binding site and an allosteric site on the clusterin molecule through hotspot analysis. We screened commercially available fragment libraries for anti-cancer activity and applied the “rule of three” to ensure drug-like properties. The highest-affinity fragment underwent “fragment-growing” to develop potential drug candidates. After docking and toxicity screening, candidate drugs were identified. Quantitative structure-activity relationship analysis revealed that the chemical size and complexity of the fragments significantly contributed to their binding affinity to clusterin. Pharmacokinetic analyses of candidate drugs, followed by molecular dynamics simulations of the top 1 final candidate demonstrated good binding affinity and significant ligand flexibility towards clusterin. Our study provides a promising approach for discovering molecules/fragments that are potential drug candidates for clusterin-induced chemoresistance.

## 1. Introduction

Cancer is one of the diseases of modern times that is difficult to treat [1]. Lifestyle, genetics, and environmental factors led to projections from demographics in the year 2022 that by 2030, cancer cases are expected to reach 35 million, with no signs of stopping [2]. Chemotherapy is the most commonly used method to treat cancer, involving chemicals such as vincristine, vinblastine, and gemcitabine to induce apoptosis through cellular stress and suppress cell defense [3]. However, problems such as fatigue, hair loss, nausea, vomiting, infertility, diarrhea, heart conditions, as well as memory and cognitive issues are just a few of the side effects commonly experienced by patients undergoing treatment [4]. According to the log-kill hypothesis, chemotherapy drugs eliminate only a fixed percentage of cancer cells. This implies that cancer cells may develop chemoresistance and even evolve to advanced stages [5]. This is part of the bigger issue of chemoresistance in cancer cells wherein cancer cells gain resistance to chemotherapeutic drugs. There are many proteins involved in chemoresistance in cancers—one protein of notable interest is clusterin (CLU), also known as lipoprotein J, a protein strongly associated with chemoresistance and considered one of the potential targets for treatments across various cancer cell lines [6]. In normal functioning cells, it is responsible for neutralizing misfolded proteins, acting as a chaperone molecule. It is also responsible for numerous physio-pathological processes, including lipid transport, cell adhesion and aggregation, complement inhibition, sperm maturation, as well as anti-apoptosis [7]. There are two forms of CLU, both of which are responsible for seemingly opposite functions. The nuclear form (nCLU), a 49 kDa protein, has been found to participate in pro-apoptotic pathways [8]. On the other hand, the secreted form of CLU (sCLU), approximately 80 kDa, have been discovered to be involved in anti-apoptotic activities [9]. In this context, sCLU has been identified as one of the key factors responsible for the chemoresistance of various cancer cell lines. sCLU was found to be abnormally represented in various cancer cell lines, including skin, pancreatic, breast, colon, esophageal squamous, lung, and neuroblastoma [10]. It was also found that the interplay between the expressions and functions of both forms of CLU changes depending on cancer progression [11]. Additionally, clusterin is also involved in the progression and action of other conditions, particularly in neurological diseases, such as Alzheimer’s disease [12] and Huntington’s disease [13], among other conditions. The most common form of treatment for abnormal CLU expression involves the use of the drug Custirsen, also known as OGX-011 (where OGX is the acronym for the company OncoGenex Technologies and 011 is the compound’s code). This drug is a second-generation antisense oligonucleotide that inhibits CLU expression and is currently undergoing phase II clinical trials, as it has been found to resensitize cancer cells to chemotherapeutic agents [14]. Despite CLU having an established treatment through OGX-011, it still has its drawbacks, namely, restricted drug delivery options, a lack of understanding of the required level of regulation adjustment to sufficiently affect the expression back to normal levels, genetic alterations leading to loss of adhesion to the target strand, and delayed onset of treatment efficacy, among other limitations [15]. Such additional treatments for dealing with elevated CLU expression levels would prove very helpful. In this study, we present potential drug candidates targeting CLU using fragment-based drug discovery (FBDD) as potential effectors for resensitizing cancer cells to chemical treatments. Other sites of interest were also determined in addition to the main binding site through hotspot searching. Qualitative structure-activity relationship (QSAR) analysis was also conducted to determine the structural properties relevant to designing effective drugs that target sCLU. Finally, molecular dynamics was investigated to determine the accurate binding energies as well as the structural stability of the complex, ligand, and protein.

## 2. Materials and Methods

### 2.1. Obtaining Crystallized Protein

The protein structure used in the study was retrieved from the RCSB Protein Data Bank (PDB) under entry number 7ZET [16]. This crystal structure was obtained by X-ray diffraction at a resolution of 2.80 Å. A ligand, 2-acetamido-2-deoxy-β-D-glucopyranose (NAG), is also present in the structure. In the protein preparation, the water molecules and ligand molecules were removed. Afterward, the missing atoms of the residues were filled in, with only the polar hydrogens added to the structure. Once the structure was complete, Kollmann charges were added, and the distribution was equalized among all residues. The resulting structure was then exported in pdbqt format, and this was used for both hotspot searching and all the docking results for both fragments and the candidate drugs. This was conducted in AutoDock Tools version 1.5.7 (from https://ccsb.scripps.edu/mgltools/, accessed on 2 December 2024) software [17].

### 2.2. Fragment Database Formulation

A fragment database was compiled from seven commercially available fragment libraries: Chembridge (https://chembridge.com/), ChemDiv (https://www.chemdiv.com/), Enamine (https://enamine.net/), FCH Group (http://fchgroup.net/), Life Chemicals (https://lifechemicals.com/), Otava (https://otavachemicals.com/), and SelleckChem (https://www.selleckchem.com/). The software Instant JChem (version 24.3.3, 2024; ChemAxon, http://www.chemaxon.com) [18] was used to apply the rule of three [19], with minor exceptions allowed to retain potentially valuable fragments that slightly violated the rule. The following filtering parameters were used: Molecular weight < 300.50 Da, tPSA < 60.50 Å, cLogP < 3.50, number of H-bond acceptors ≤ 3, number of H-bond donors ≤ 3, and number of rotatable bonds ≤ 3. Anti-cancer ability was then evaluated using pdCSM-cancer (from https://biosig.lab.uq.edu.au/pdcsm_cancer/) prediction [20] (accessed on 11 December 2024). The fragments that were labeled as active in the general cancer activity results were chosen for the final database.

### 2.3. Molecular Docking Procedure

Prior to docking, ligands were prepared using OpenBabel version 2.4.0 [21,22] to add hydrogen atoms and generate 3D structures. PyRx version 0.8 [23] was then used to perform energy minimization using the Universal Force Field (UFF) and to convert the ligands into .pdbqt format. Blind docking of the final fragment database was conducted across the entire protein surface using AutoDock Vina [24,25]. To identify potential binding sites. The docking protocol was validated by redocking the co-crystallized ligand (NAG) into the protein structure. The accuracy of the docking protocol was assessed by calculating the Root Mean Square Deviation (RMSD) between the re-docked and original ligand poses.

Binding regions identified from the docking results were further supported through hotspot analysis using FTMap (https://ftmap.bu.edu/; accessed on 2 December 2024) [26,27]. The same parameters and workflow were subsequently used for docking the candidate compounds derived from the fragment-growing approach.

### 2.4. Fragment Growing

The fragment with the highest binding affinity was selected for expansion using the FragGrow web server (https://fraggrow.xundrug.cn/; accessed on 12 December 2024) [28]. Two growing modes were considered in this step: direct growing by replacing hydrogen atoms or substructures, and virtual synthesis through retrosynthesis-based replacement. This procedure generated a total of 7499 candidate molecules based on the top fragment.

### 2.5. Candidate Drug Filtering Using T.E.S.T Software

To ensure that only non-toxic and non-mutagenic compounds were retained, the Toxicity Estimation Software Tool (T.E.S.T) version 5.1.2 (https://www.epa.gov/comptox-tools/toxicity-estimation-software-tool-test) (accessed on 28 December 2024) [29] was used. The following parameters were predicted using the nearest-neighbor method: Fathead Minnow LC_50_ (96 h), *Daphnia magna* LC_50_ (48 h), *Tetrahymena pyriformis* IGC_50_ (48 h), oral rat LD_50_, bioconcentration factor, developmental toxicity, and mutagenicity. Only compounds predicted as both “Developmental Non-toxicant” and “Mutagenicity Negative” were selected for further analysis.

### 2.6. Quantitative Structure-Activity Relationship of Candidate Drugs

Further evaluation of the structural features and their correlation with binding affinity was performed using Quantitative Structure-Activity Relationship (QSAR) analysis [30]. Molecular descriptors were extracted using the RDKit Python library (version Release_2024.09.4) [31] and modeling was conducted using Scikit-learn’s MLPRegressor version 1.6.1 [32] with an artificial neural network architecture. The primary predictor variable was the binding affinity obtained from the docking experiments. A 5-fold cross-validation approach was employed to assess model performance. Metrics such as R^2^ for training and test sets, along with normalized RMSE, were used for evaluation. The applicability domain was analyzed using Principal Component Analysis (PCA) optimized by t-SNE.

### 2.7. Absorption, Distribution, Metabolism and Excretion (ADME) Filtering of Candidate Drugs

The ADME properties of the remaining candidates were evaluated using the SwissADME web server (http://www.swissadme.ch/; accessed on 24 January 2024) [33]. Compounds were filtered according to the following criteria: (I) ESOL and Ali class: at least “moderately soluble”; (II) Not permeant to the blood-brain barrier (BBB); (III) Not inhibitors of CYP1A2 and CYP3A4; and (IV) Minimal violations of drug-likeness rules. This filtering yielded two final candidate drug precursors, one of which was selected as the top candidate based on its lipophilicity, which is related to the study’s objective of targeting extracellular sCLU.

### 2.8. Molecular Dynamics Simulation Procedure

Molecular dynamics (MD) simulations were conducted using GROMACS version 2024.4 [34]. Ligand and protein preparation was performed with the BioBB Python module version 5.0.0 (2024.2) [35] using the AMBER force field [36] for both molecules. The system was solvated and sodium and chloride ions were added for charge neutralization. Equilibration was carried out under NVT and NPT ensembles to stabilize temperature and pressure. The production MD simulation lasted 100 ns, using a 2 fs time step for a total of 50 million steps. The RMSD, RMSF, and radius of gyration (Rg) were calculated to assess the structural stability of the complex, protein, and ligand. Binding free energy analysis was conducted using the MM-GBSA approach [37] via the gmx_MMPBSA Python tool version 1.6.4 [38]. The following components of the binding energy were computed: van der Waals (VDWAALS), Electrostatic (EEL), Polar Solvation (EGB), and Non-polar Solvation (ESURF). Convergence analyses were also conducted to determine if the results eventually stabilized over the course of the simulation. Convergence plots were created along with block averaging and moving window analysis to reduce the noise and illustrate the standard deviations. The Augmented Dickey-Fuller (ADF) test [39] was also conducted to statistically determine if the values did indeed converge into a stable value. In total, four independent molecular dynamics simulations were conducted for each ligand.

## 3. Results

### 3.1. Fragment Database Features and Composition

**Figure 1:**
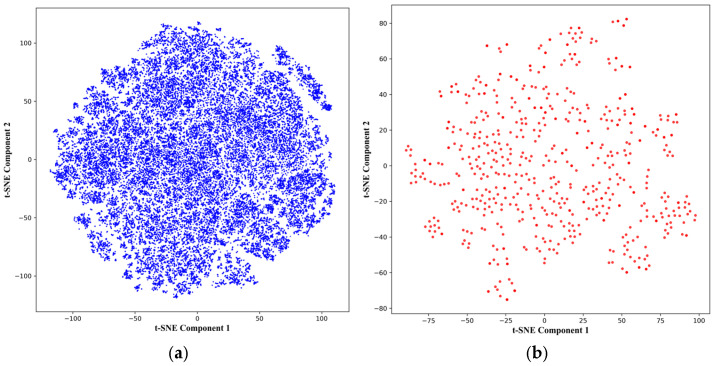
t-SNE-aided PCA plots displaying the chemical space covered by both databases: (**a**) The main fragment database prior to cancer activity filtration and (**b**) fragment database with only active anti-cancer activity, as predicted by pdCSM-cancer.

The fragment database underwent two different filtration steps. The first step involved removing compounds that did not meet the thresholds set by the rule of three [19], as well as removing duplicates across databases. From a total of 60,834 compounds across all databases, the first filtration resulted in a filtered database containing 58,937 fragments. Afterward, the cancer activity filtration step was conducted using the pdCSM-cancer [20], the final fragment database used in this study, which was composed of 607 predicted cancer active fragments. To describe the amount of chemical space covered by the fragment databases used in the study, PCA combined with t-SNE dimensionality reduction was used to describe the chemical spaces covered by the databases. Under t-SNE-aided [40] principal component analysis [41], the plots presented in Figure 1 represent 43.44% and 66.33% of the variance of the main fragment database and the final fragment database. The normalized RMSD values for the main and final fragment databases were 0.0364 and 0.0325, respectively. This indicates generally low variability across both databases. This can be explained by the fact that both databases are based on the definition of the rule of three, as well as commercially available databases having inherently similar compounds according to the specific categories such databases offer, resulting in lower chemical variability. It can also be seen that the coverage of chemical space decreases after cancer activity filtration. This is because pdCSM-cancer evaluates cancer activity on QSAR models built on many cancer cell lines; this favors certain chemical and structural properties, thereby further reducing chemical space coverage [20]. Table 1 lists 10 example fragments that passed both filters. The full final fragment dataset is available in Supplementary Materials Table S1.

### 3.2. Blind Docking of Cancer Active Database and Validation of Hypothesized Binding Site Through Hotspot Analysis

**Figure 2:**
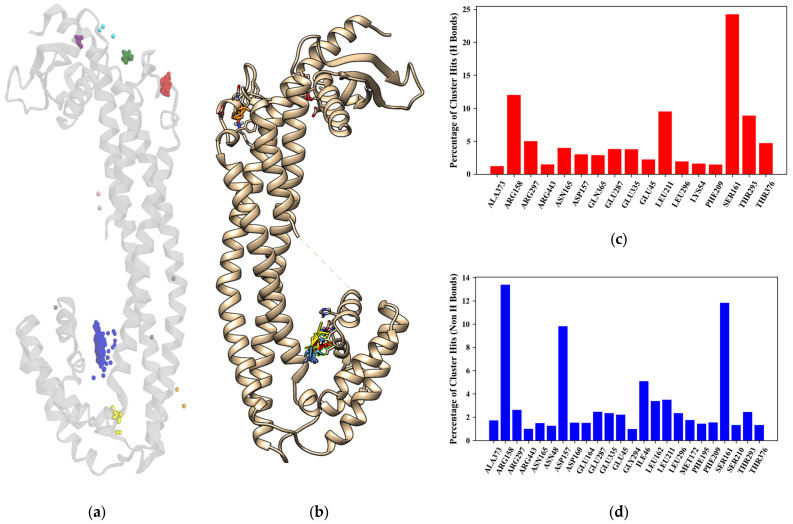
Visualizations of the fragment docking distribution across the entire sCLU structure. Each color represents a different cluster according to DBSCAN (**a**), FTMap results (**b**) (illustrated using UCSF Chimerax developed by the Resource for Biocomputing, Visualization, and Informatics at the University of California, San Francisco, with support from the National Institutes of Health R01-GM129325 and the Office of Cyber Infrastructure and Computational Biology, National Institute of Allergy and Infectious Diseases [44]), and the interaction hits for both (**c**) H-bond and non-H (**d**) bonds show close correlation with the results from the docking experiment. This, along with the previous results, showed the potential main catalytic site and possible allosteric sites of clusterin.

As the specific binding site for sCLU is currently undetermined in the literature, the docking conducted in this study was blind docking. As such, all the fragments from the final fragment database were docked onto the entire surface of sCLU. The results are illustrated in Figure 2a, showing the clusters found by Density-Based Spatial Clustering of Applications with Noise (DBSCAN) [42]. Docking validation resulted in a mean RMSD of 2.31 Ångströms; this suggests that the docking predictions produced acceptable results when compared to real-life experiments [43]. The clustering procedure produced nine distinct clusters whose constituents were within 5 Ångströms from each other. The population of each cluster and the predicted interacting residues within 3.5 Å of the compounds are presented in Table 2.

It can be seen that the majority of the compounds docked onto the binding pocket on the latter portion of the protein, as shown in both Figure 2a and Table 2. This is captured in the blue cluster, which shows that the residues GLU164, ASN165, HIS290, THR293, ASP157, ARG158, GLU335, PHE143, ASP160, ARG297, GLU287, TYR151, ASN291, and SER161 show the highest number of interactions with docked fragments. These results were corroborated with the results from FTMap [27], which are shown in Figure 2b.

**Figure 3:**
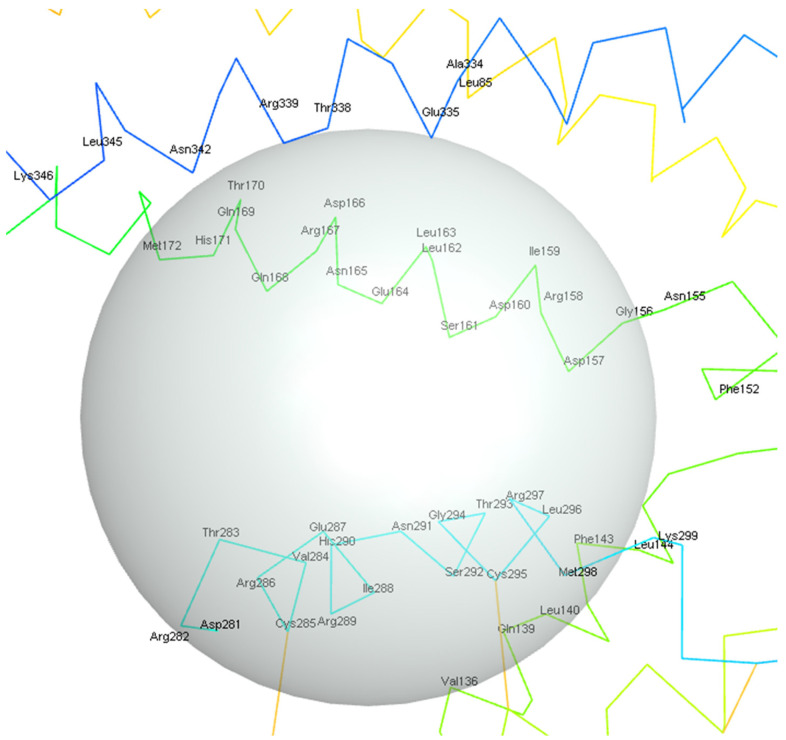
Illustration of the main binding site, enclosed in the gray sphere, according to the number of cluster hits in FTMap and the docking experiment (illustrated using Biovia Discovery Studio [45]).

Figure 2c,d shows the residues contributing most to the hotspot analysis. Of note are the residues SER161 and ARG158, both of which were determined to have a large number of fragment cluster hits for both H-bonds and non-H bonds (the raw results from FTMap are available in Supplementary Materials Table S2). In Figure 3, the potential binding pocket that contains all relevant residues, as indicated by both the docking procedure and hotspot analysis, is shown.

There are three main helices that contribute to the binding site of the protein. This is the only region of the protein where this helical structure appears, suggesting a specialized functional role. In addition, the major residues highlighted in Figure 2c and in Figure 2d, such as ARG158, ASP157, SER161, and THR293, were found to be present within this proposed main binding site. The only exceptions are the residues associated with the areas around ILE46 and LEU211, which are all located on the opposite side of the protein. As sCLU has been determined to display allosteric binding capability [46], these sites may offer future targets for clusterin inhibition. These sites are also shown in Figure 2b as well as in Figure 2a, which may be used as references for allosteric site targeting experiments.

**Figure 4:**
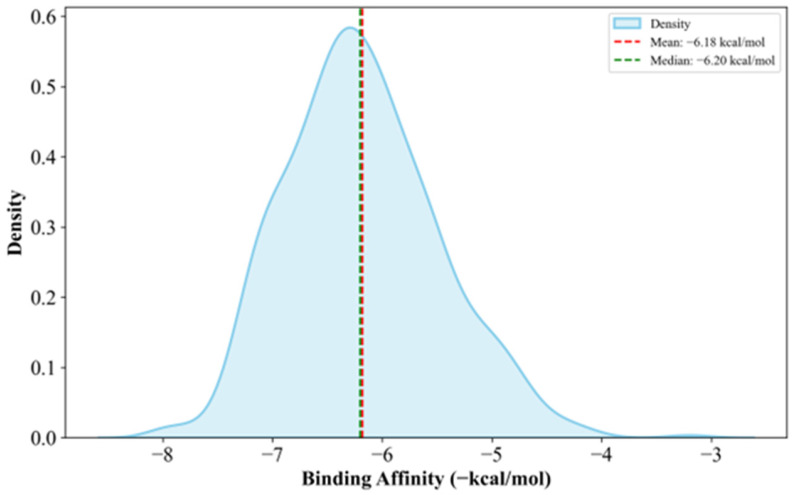
Fragment binding efficiency distribution (visualized using the Seaborn Python library ver. v0.13.2 [48]).

The docking results showed a relatively normal distribution, as shown in Figure 4, with docking scores ranging from −8.0 kcal/mol to −3.2 kcal/mol. As shown in Figure 4 and Table 3, the docking scores tend to show relatively low affinity to the protein; this is expected in FBDD, where fragments are small and have limited functional groups, often resulting in lower docking scores. This is because smaller fragments have less surface area and fewer functional groups, which tend to further improve docking scores [47]. The full dataset containing the binding efficiencies of all docked fragments is available in Supplementary Materials Table S3.

**Figure 5:**
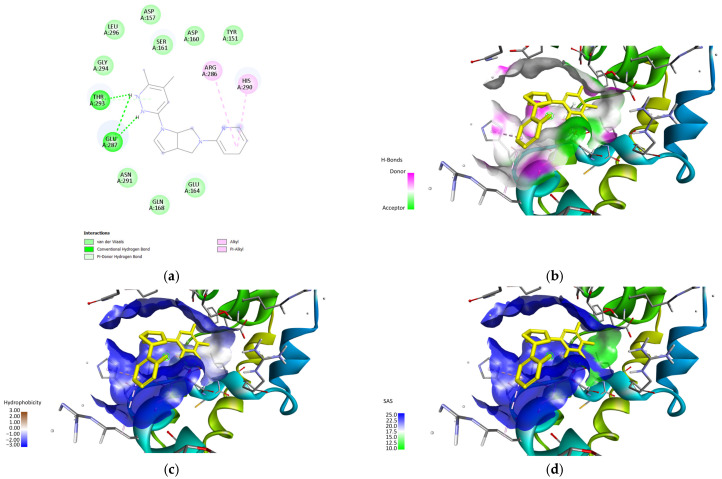
2D illustration of the interactions (**a**) and the H-bond (**b**), hydrophobicity (**c**), and SAS (**d**) surface found in the binding site of the top 1 fragment hits (illustrated using Biovia Discovery Studio [45]).

Figure 5 shows the docking pose of the top 1 fragment in Table 3. This illustrates the reason for the lower affinity scores, with only four residues interacting and only THR293 being part of the major residues previously established in Figure 2c,d. It can also be noted that the binding pocket is highly hydrophilic, supported by a relatively high SAS score. This supports the proposed function of this region as a potential binding site as these qualities are generally correlated with signaling proteins, which is one of the functions of clusterin [49,50]. Such results may also suggest a highly flexible region of the protein which may be involved in various unstressed ligand interactions, which are also recorded in the literature [46].

### 3.3. Fragment Growing and Docking of Candidate Drugs to Clusterin

**Figure 6:**
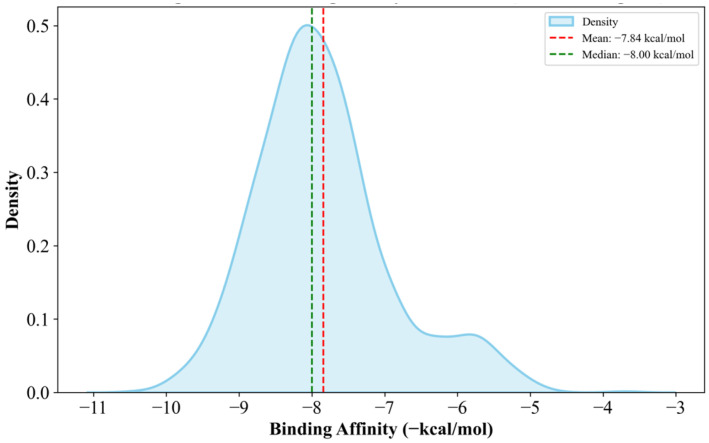
Distribution of docking scores of candidate drugs (visualized using Seaborn Python library ver. v0.13.2 [48]).

Fragment growing involves adding or replacing certain structures onto a base compound to improve certain capabilities of a compound. In this study, only the fragments with the highest affinity to the protein were taken for fragment-growing. This is because the program used (more information provided in Section 2), offered many options to further optimize the protein, including replacement of substructures that make it similar to other fragments, as well as growing through H replacement at various points of the fragment. The resulting 7499 compounds were filtered down into 194 candidate drugs for clusterin, using non-toxicant and non-mutagenic filters. These are recorded along with their affinity and T.E.S.T. [29] toxicity-predicted values in Table 3. The distribution of binding scores is also shown in Figure 6.

The fragment-growing resulted in a significant improvement in the binding affinity of the candidate drugs. This is due to the larger amount of surface area and added functional groups because of the growing procedure. The mean docking score increased by 26.82% and the new highest affinity candidate drug had a docking score of −10.4 kcal/mol. It should also be noted how some variants of the fragment growing decreased the docking score from the baseline of −8.0 kcal/mol. These were found to be mostly due to some entries undergoing substructure replacement; this may result in substructures with lower affinities than the established base structure of the fragment.

In addition to the docking affinities, the toxicity of the compounds should also be considered. The predicted toxicity values of *Daphnia magna*, Fathead minnow, and *T. pyriformis* (see also Table 4) cell lines show an inverse relationship—the lower the predicted values, the higher the predicted toxicity to the aforementioned cell lines [51]. Candidates such as 0000625345lig, 0000437319lig, and 0000608089lig showed high potential for bioaccumulation. In addition, 0000874651lig, 0000750663lig, 0000588427lig, and 0000957253lig showed high predicted toxicity to *Daphnia magna.* For the other aquatic toxicity parameters, all the top 10 ligands showed high potential toxicity to Fathead cell lines. *T. pyriformis* predicted toxicity involved several candidates which showed relatively high toxicity, such as 0000588427lig, 0000874651lig, 0000514254lig, 0000345617lig, and 0000608089lig. In terms of oral rat LD50, all candidates were able to show relatively low predicted oral toxicity to rats as shown by the high oral rat LD50 values [52]. Although these values cannot be directly correlated with toxicity in humans, they are indicative of the need to further test the presented candidate drugs for their toxicity in actual experiments. However, what can be suggested is that some of the candidate drugs display significant potential environmental risks, which should be considered in actual applications of these candidate drugs. Complete docking scores of all candidate drugs, along with their T.E.S.T. predicted toxicities are available in Supplementary Materials Table S4.

**Figure 7:**
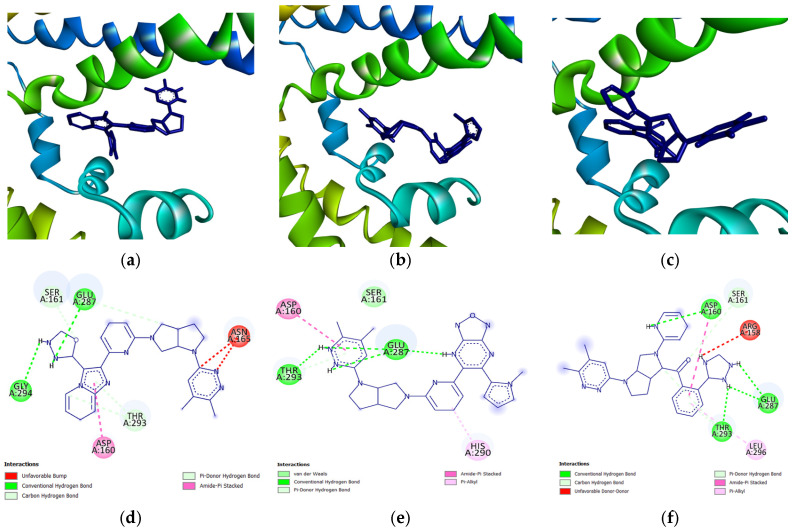
Docked poses of the top 1, top 2, and top 3 potential candidate drugs (**a**–**c**) and 2D illustration of the interactions found in each respective ligand (**d**–**f**). (illustrated using Biovia Discovery Studio [45]).

To further identify the factors improving the docking score of the candidate drugs from the reference fragment, Figure 7 shows the protein-ligand interactions of the top three candidate drugs, as shown in Table 4. The first noted additions to the participating residues are the main contributors shown in Figure 2c,d. Residues such as SER161 and ARG158 were now active in the binding of the candidate drugs to the protein; this is the result of hydrogen replacement by functional groups. It can also be noted how much larger the candidate drugs are when compared to the original, as well as how the shape and 3D conformation of the candidate drugs fit around the binding site; this illustrates one of the main advantages of FBDD compared to other drug discovery pipelines [47].

Although these improvements to the binding affinity are significant, it should be noted that only one round of fragment-growing was conducted; as such, it is yet to be determined what effects more stages of growing optimization would have in this case. There is the potential problem of over-fitting a potential drug to a stationary protein which is the case in the docking experiment. Proteins commonly shift their structures according to certain stimuli; in such cases, some drugs may prove ineffective at binding onto the target. It is for this reason that allosteric site targeting, which is also possible through FBDD, is highly recommended as an avenue for the creation of new drugs [54].

### 3.4. Quantitative Structure-Activity Relationship Model Development, Performance, and Implications

**Figure 8:**
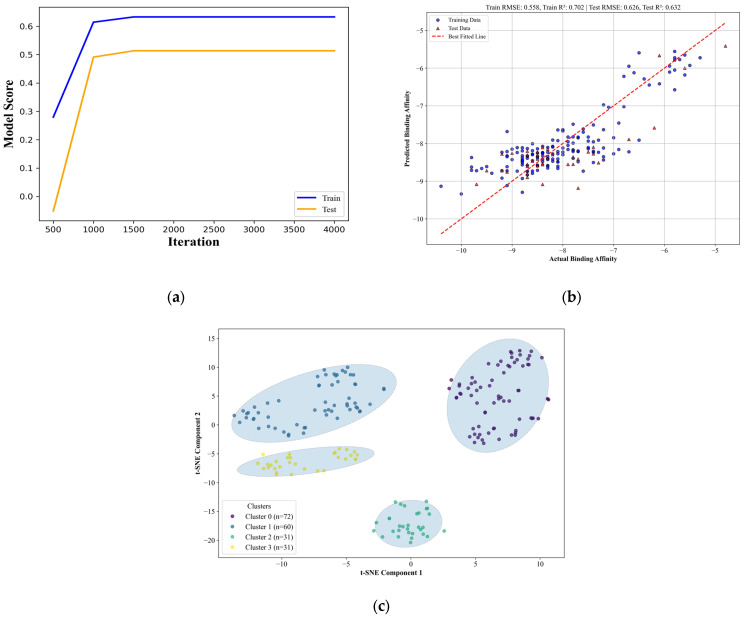
The iteration learning curve of the ANN model indicates convergence of scores at 1500 iterations (**a**). The parity plot of QSAR model prediction vs. actual binding efficiency shows good potential predictive and descriptive ability (**b**). On the other hand, the chemical space coverage showed four distinct clusters according to 2D and 3D chemical and structural descriptors that are within the applicability domain of the QSAR model (**c**). Clustering for the applicability domain was conducted through DBSCAN [42].

To determine the contributions of certain descriptors to the binding affinity, QSAR was conducted using an artificial neural network (ANN). In the model optimization process, it was determined that an iteration count of 1500 was required for the models to properly converge; the learning curve is shown in Figure 8a. Different combinations of hidden layer sizes were tested. Because of computational and time resource limitations, only three hidden layers were assigned to the model and all combinations of layer sizes for all three layers, ranging from 10 to 200 in increments of 10 neurons, were tested. Table 5 shows the highest-ranking models as well as their associated scoring parameters.

From Table 5, it is apparent that in some metrics, the model did not completely meet the criterion for good predictive ability, which is that the normalized RMSE should be less than 10% for it to be considered a good predictive model. However, it should be noted that both the test score and cross-validation mean gave support to the model’s capability to describe the phenomenon; this can be further supported by the cross-validation score, which, while generally observed in this case to be lower, is still within a close range of the test scores, indicating good consistency in its predictive ability. Given that the normalized RMSE is not far from the threshold and the test scores in conjunction with the cross-validation imply giving credence to the model, it can be said that the model is adequate to describe the relationships between the structural and chemical descriptors and the binding efficiency of each candidate drug [55]. Supplementary Materials Table S5 shows all the test scores for the remaining models. The parity plot shown in Figure 8b also supports this conclusion as the predicted and actual values still display a relatively high degree of linearity, further supporting the model’s accuracy.

In terms of the descriptors, it became apparent that some descriptors contribute more to the predictions than others. As seen in Table 6, the descriptor with the highest contribution to prediction is the molecular complexity, as evaluated using AvgIpc, followed by Balaban’s J value, which is also related to the structure, specifically, quantifying the degree of branching [31]. The rest of the descriptors have a relatively minor influence on the model’s performance. These results suggest that the individual variations in terms of small substructures matter less to the performance of the model when compared to the overall structure of the compound. In other words, it can be said that the specific functional groups matter less in affecting the docking affinity than the overall complexity of the structure. This can be better seen with the applicability domain of the QSAR model, as shown in Figure 8c. The clusters shown in the figure, which account for 77.03% of the variance, show how closely related the clusters are in terms of structural descriptors. Clusters 3 and 1 are more closely related to each other than to cluster 0, and all three clusters are more closely related to each other than to cluster 2. Table 7 shows the mean and standard deviation of each relevant physical feature that distinguishes the four clusters. It can be seen that the molecules in cluster 2 are generally smaller and can be inferred to be less complex than the other clusters, as shown by the lower molecular weight, volume, surface area, and subsequent binding affinity. Table 7 also shows in quantitative terms the close relationship between clusters 3 and 1 as their physical descriptors are close to each other. The same can be said for the collective cluster of 3 and 1’s relationship with cluster 0, which can also be seen to be close to the two clusters though generally more distant.

The clusters are more apparent when considering the structural features of the members. In Supplementary Materials Figure S1, the members of cluster 0 are primarily composed of candidates which are the result of replacements of the hydrogens on the peripheral pyridine and the pyridazine substructures. Clusters 1 and 3 both feature results of hydrogen replacements as well; however, these clusters involved replacements in the central octahydropyrrolo[2,3-c]pyrrole analog. On the other hand, cluster 2 contains the results of the substructure replacement. As is apparent from the cluster relationships shown in Figure 8c, the differences between clusters 1 and 3 are minimal, with few distinguishing features between the two.

### 3.5. ADME Properties Reveal Potential Final Drug Candidates for Non-Brain-Related Cancers

**Figure 9:**
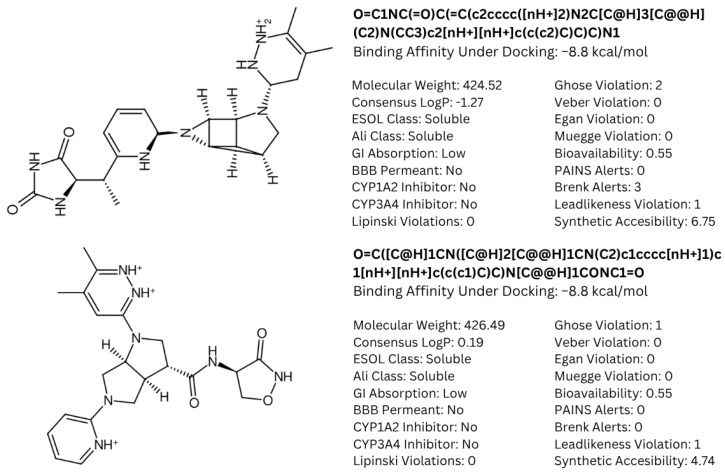
Structures of cluster members at each 20th percentile in terms of binding efficiency. Structures illustrated by SMILES to Structure [53].

To further classify the candidate drugs into some potential areas of application according to their pharmacokinetic properties, ADME analysis was conducted using the SwissADME webserver [33] on the candidate drugs whose binding affinities were higher than the binding affinity of the parent fragment. Of note are the results for the inhibitor and substrate of the highlighted enzymes relevant to drug absorption and BBB activity. In this study, the main filtering criteria were set to only include candidate drugs that are non-BBB permeant, not inhibitors of CYP1A2 and CYP3A4, and whose ESOL class and Ali class, indicating water solubility and lipophilicity, respectively, do not violate any criterion for drug compounds of baseline moderate solubility. These filtering criteria select candidate drugs that can be applied for non-neurological-related cancers using the BBB permeant filter, whilst accounting for proper absorption through the CYP1A2 [56] substrate violation filter. Additionally, potential cytotoxic clashes with other drug effects are also mitigated by filtering for CYP3A4 inhibition [57]. Figure 9 lists the candidate drugs that passed the ADME filters; these candidate drugs were then considered to be the final drug candidates.

While these two drugs were selected as the primary candidate drug precursors or templates for our study, other potential candidates and their ADME analysis outputs are detailed in Supplementary Materials Table S6, recognizing their potential utility under different circumstances. The final candidate drug on the left side of Figure 9 shows higher lipophobicity compared to the other, and this was the drug chosen for molecular dynamics analysis. This research assumes that the clusterin target is in its secreted form, which is sCLU [58]. Future studies targeting intracellular proteins or proteins involved in the CNS, such as nCLU, may require modifications of the candidate drugs which were curated to fit criteria for both pharmacodynamics and pharmacokinetics studies. Further investigations, encompassing both in vitro and in vivo studies, could be conducted to identify and address the limitations of our current research.

### 3.6. Molecular Dynamics Simulation of Top 1 Final Drug Candidate

**Figure 10:**
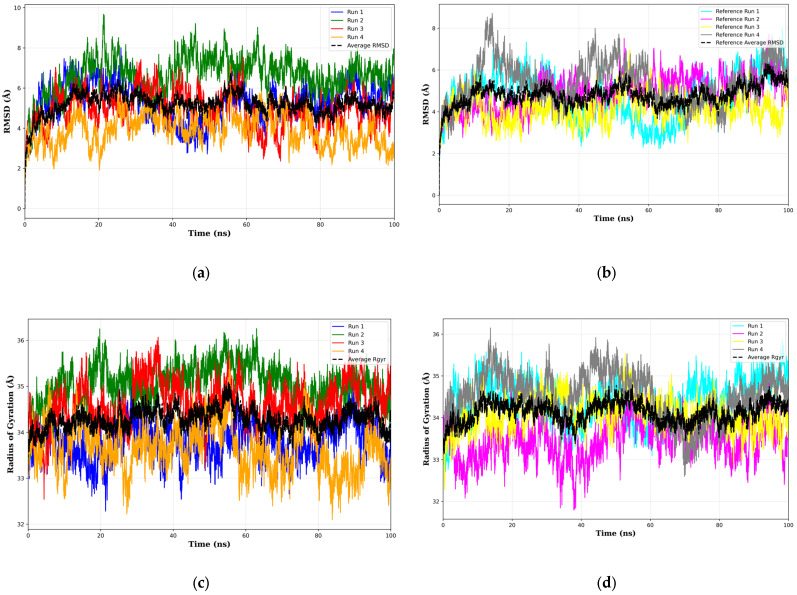

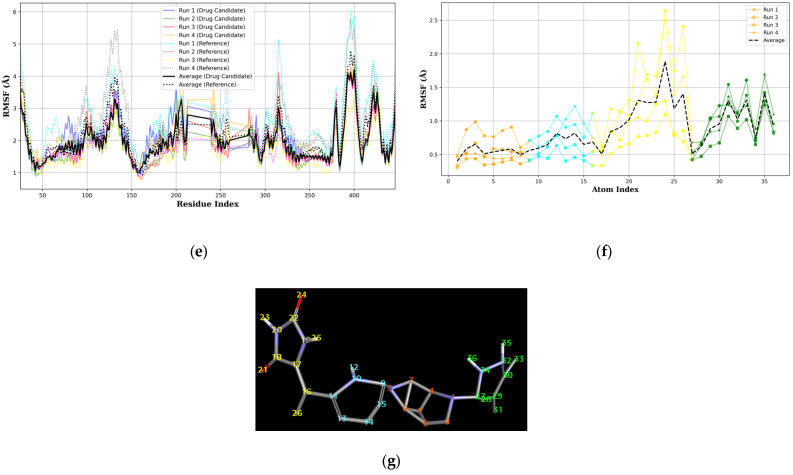
Results of RMSD compared to first frame of the top 1 final candidate drug precursor (**a**) and reference ligand (**b**), radius of gyration of the top 1 final candidate drug precursor (**c**), radius of gyration of the top 1 final candidate drug precursor (**d**), RMSF of the protein across all runs (**e**), RMSF of the top 1 final candidate drug precursor colored according to substructure (**f**), and the 3D representation of the top 1 final candidate drug precursor with the atom indices labeled and colored according to results of RMSF analysis (**g**). Illustrated in Biovia Discovery Studio [45].

To further evaluate the binding affinity of the top-ranked final drug candidate, as determined by both ADME filtering and final docking, molecular dynamics (MD) simulations were performed. Root mean square deviation (RMSD) analysis was performed to assess the structural deviations throughout the simulation relative to the initial frame. A total of four runs were conducted for both the top 1 final drug candidate and the reference ligand, NAG. All the runs were conducted with different initial velocities and different conditions, the details of which are detailed in Supplementary Materials Table S7. Stability was assessed using the radius of gyration. To assess the conformational changes, root mean square fluctuation (RMSF) analysis was also conducted for both the protein backbone and the ligand; the latter was only conducted for the drug to determine the substructures that tend to move around in the simulation. Figure 10 shows the results of the molecular dynamics simulation for both the top 1 final drug candidate and the reference ligand as they relate to structural stability and mobility.

Comparing the RMSD values from Figure 10a,b shows remarkably similar means for the RMSD values over their respective runs: 5.19 Å (complete data available in Supplementary Materials Table S8) and 4.80 Å (complete data available in Supplementary Materials Table S9) for the top 1 final candidate drug precursor and the reference ligand, respectively. Convergence analysis reveals that under the cumulative average plot for the top 1 final candidate drug precursor, Supplementary Materials Figure S2, and the reference ligand, Supplementary Materials Figure S3, stabilization starts in the time range of 20 ns to 40 ns. Block averaging analysis and moving window analysis also shows a consistent moving standard deviation across the entire run time of the simulation. These can be seen in Supplementary Materials Figures S4 and S5 for the top 1 final candidate drug precursor, and Supplementary Materials Figures S6 and S7 for the reference ligand. The ADF test results (Supplementary Materials Table S10) show highly negative ADF statistics and *p*-values less than the 0.05 threshold. This indicates strong evidence for stabilization with regard to the RMSD of both ligands. The radius of gyration results in Figure 10c (mean of average radius of gyration = 34.28 Å) and 10d (mean of average radius of gyration = 34.12 Å) are similar; data for the top 1 final candidate drug precursor and the reference ligand are available in Supplementary Materials Tables S11 and S12, respectively. The convergence of the radius of gyration was also tested. The cumulative average plots shown in Supplementary Materials Figures S8 and S9 for the top 1 final candidate drug precursor and the reference ligand, respectively, show relatively unstable plateaus. Regardless, the ADF test results (Supplementary Materials Table S10) once again showed strong evidence for stabilization across all runs with regard to the radius of gyration. The block averaging plots for the top 1 final candidate drug precursor and the reference ligand can be seen in Supplementary Materials Figures S10 and S11, respectively. In addition, Supplementary Materials Figures S12 and S13 show the moving window plots for each ligand. RMSF analysis of the protein, seen in Figure 10e, reveals that the stabilization of the protein backbone is relatively similar in both complexes. Summary statistics of the RMSD and the radius of gyration are shown in Supplementary Materials Table S13.

**Figure 11:**
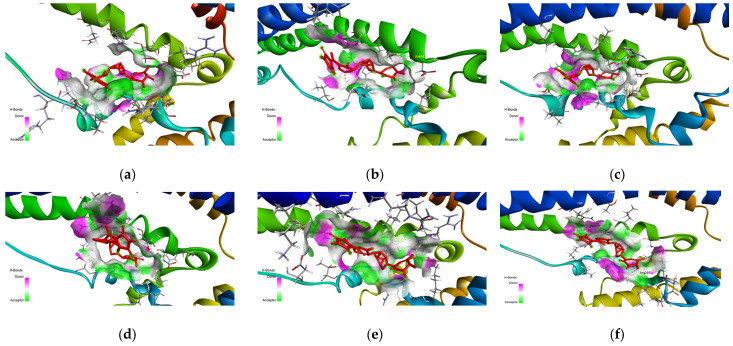
Illustrated poses of the CLU-Top 1 Final Candidate Drug complex at 0 ns (**a**), 20 ns (**b**), 40 ns (**c**), 60 ns (**d**), 80 ns (**e**), 100 ns (**f**). Illustrated using Biovia Discovery Studio [37].

Although the RMSD values are roughly similar between the two complexes, a possible explanation is that due to the increased number of rotational bonds, size, and complexity of the top 1 final candidate drug precursor, the gyration of the drug results in a higher RMSD as certain substructures move around within the binding pocket. This can be seen in Figure 10f,g, where the peripheral substructures (atoms labeled in green and yellow) have higher RMSF values than the core substructures (atoms labeled in cyan and orange). This can also be seen in Figure 11, which shows the structure of the CLU-Top 1 Final Candidate Drug Precursor complex at various points in the first molecular dynamics simulation. Additional figures showing the remaining runs of the sCLU-Top 1 Final Candidate Drug Precursor complex are shown in Supplementary Materials Figure S14.

**Figure 12:**
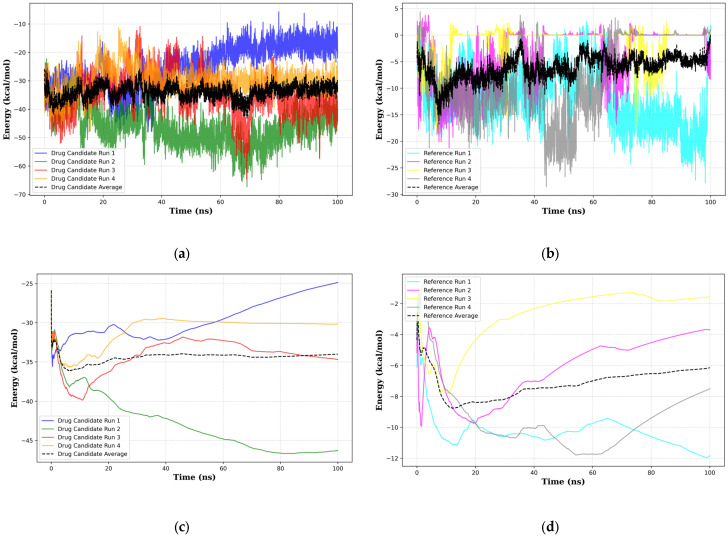
Plotted GB delta total, in kcal/mol of the top 1 final candidate drug precursor complexes (**a**) and the reference ligand (**b**) and cumulative average energies for both the top 1 final candidate drug precursor (**c**) and reference ligand (**d**).

Molecular mechanics with generalized Born and surface area solvation (MMGBSA) analysis was conducted to evaluate the total delta energy of the system. This was to determine the binding affinity of the top 1 final candidate drug precursor compared to the reference ligand. Figure 12 shows the results of the MMGBSA analysis.

Figure 12a and Figure 12b show the GB delta total for both the top 1 final candidate drug precursor and the reference ligand (remaining energy term plots are available in Supplementary Materials Figures S15 and S16, respectively). What is immediately apparent is that the average of the latter represents a generally higher affinity than the former. Cliff’s delta analysis for all energy terms shows a Cliff’s delta value of −1.0, indicating a large effect size with a highly significant difference across all energy constituents. Similarly, with the RMSD and the radius of gyration, cumulative average plots of the GB delta compounded with additional cumulative average plots for van der Waals, electrostatic, polar solvation energy, and non-polar solvation energy for the top 1 final candidate drug precursor and the reference ligand (Supplementary Materials Figures S17 and S18) show that some runs achieve stable convergence whereas others are visually uncertain. Similar observations can be made for the block average analysis (Supplementary Materials Figures S19 and S20) and moving window analysis (Supplementary Materials Figures S21 and S22). The ADF test results for the top 1 final candidate drug precursor show that only one, the total GB of run 1, did not reach stability. On the other hand, nine energy terms were unable to reach stability. The data regarding the ADF analysis for the MMGBSA results can be seen in Supplementary Materials Table S14. This could imply that the sCLU-NAG complex is less stable than the relatively robust results shown in the MMGBSA analysis of the top 1 final candidate drug precursor. Summary statistics of the MMGBSA results are shown in Supplementary Materials Table S15.

## 4. Discussion

While clusterin has been identified for some time, its specific characteristics and functions are still being investigated. This includes the specific sections of the protein relevant to its interactions with ligands and residues with other proteins with respect to its main function as a chaperone protein [59]. With the aid of a fragment-based methods approach, it is possible to elucidate the dynamic functions of clusterin along with potential drugs.

The main binding site as described in Figure 3 is composed of the amino acids ARG158, ASP157, SER161, and THR293 and is of particular interest as the main ligand binding site as it can be treated as a helix-helix interface forming a pocket with an angle between the three main helix bundles and the bottom bundle. This formation has been cited in the literature as a motif with a high capability to host protein-ligand interactions, as shown in the leucine zipper nomenclature [60].

**Figure 13:**
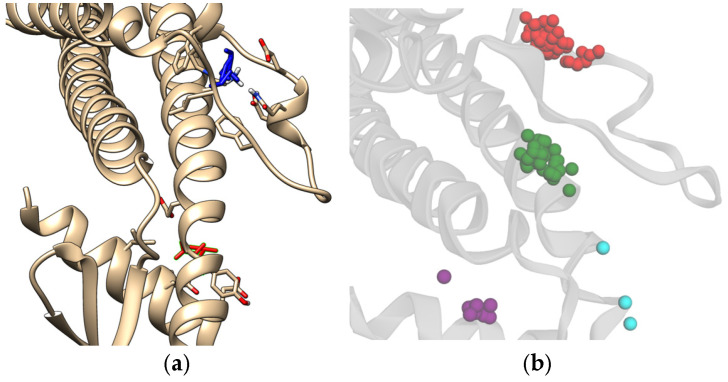
The potential allosteric site identified in FTMap (**a**) (illustrated using UCSF Chimerax developed by the Resource for Biocomputing, Visualization, and Informatics at the University of California, San Francisco, with support from the National Institutes of Health R01-GM129325 and the Office of Cyber Infrastructure and Computational Biology, National Institute of Allergy and Infectious Diseases [43]) and in the docking experiment (**b**).

Past studies on the dynamics of the structure indicate that this specific conformation is highly flexible, potentially accommodating a wide variety of ligands through allosteric or cooperative behavior [61]. This is shown through docking studies conducted as well as the literature on chaperone proteins. Another aspect to take note of is the potential allosteric sites found using FTMap, as well as in the actual docking predictions. Figure 13 shows the potential allosteric binding sites, and based on Table 2, the residues covered by the clusters are LEU211, ALA373, THR376, SER210, PRO212, PHE195, ARG194, GLN201, GLU199, LEU375, and GLY378 for the red, green, and purple clusters from the docking results. The residue interactions from FTMap also indicate LEU211, ALA373, THR376, SER210, and PHE195 residues as minor hotspots with respect to the main binding site previously described in this study. CLU has been established to display allosteric binding activity, namely, having three distinct classes of binding sites that cater to LRP-2, unstressed ligands, and stressed proteins [46]. It is also established in the literature that the LRP-2 and stressed proteins are hypothesized to bind around the C-terminus of the alpha chain, which is also adjacent to the N-terminus of the beta chain, a region that would roughly coincide with the hotspots, specifically, near the green and purple clusters. This also suggests that the unstressed ligands do indeed bind to the specified binding site assigned for unstressed ligands. It should be noted that further analysis of these hotspots would be required to fully confirm the region in which the protein-protein interactions of sCLU take place.

As a rule, chaperone proteins have a large range of targets they can bind to, much more so for chaperones which have complex roles in many signaling pathways, like sCLU, which is involved in pathways such as the NF-κB signaling pathway [62], the PI3K/Akt pathway [63], and the Reelin-signaling pathway [64]. This can be seen in the results of the applicability domain of the QSAR model, where four distinct chemical spaces were found to be able to properly bind to the protein with a relatively good binding score. The chemical space covered by the candidate drugs showed what can be seen as an advantage and a disadvantage of the druggability of chaperone proteins [65]. To address the concern of specificity, known anti-cancer drugs that have been approved by the FDA and EMA [66] were docked for comparison; the results are available in Supplementary Materials Table S16. Generally, the anti-cancer drugs show lower affinities to clusterin with a mean of −6.36 kcal/mol, which is 18.88% less potent than the candidate drugs, as shown in Figure 7.

Another concern is the issue of the synthesizability of the resulting candidate drugs. A concern with candidate drugs produced for highly specific proteins is when they become so highly matched to the protein that it significantly reduces the synthesizability of the drug [67]. Synthesizability analysis conducted using the DeepSA webserver (available from https://bailab.siais.shanghaitech.edu.cn/services/deepsa/, accessed on 9 January 2024) [68], shown in Supplementary Materials Table S17, supports the idea that targeting less-specific proteins may potentially result in higher synthesizability results. The analysis showed that 148 compounds have higher easy-to-synthesize than hard-to-synthesize scores. This is also due to the algorithms used as the majority of the candidate drugs are from H-direct grow replacements and retrosynthesis-based replacements [28]. This results in candidate drugs that can be easily synthesized, especially compared to compounds with alterations to the main substructures. On the other hand, the relatively low specificity of the ligands may present the possibility that the ligands may also bind to other proteins that are not the main target. This is not only potentially bad for the in vivo activity of the drug, as not only could it potentially disrupt the dosage required for appropriate inhibition of the receptor, but it may also have unintended toxic consequences on other parts of the cell [69]. This can be seen with the toxicity results from T.E.S.T., as shown in Table 4, whereby the candidate drugs produced a significant toxicity risk to certain benchmark strains—the nondescript nature of the compounds implicated them according to some structural qualities deemed toxic by the T.E.S.T. predictive models [29].

Currently, fragment-based drug candidates remain amenable to further optimization. Candidate drugs may be functionally modified to become more specific and thus obtain higher binding affinities through further optimization of the fragment growing process as well as by implementing other fragment optimization strategies, such as fragment reduction, to remove any unwanted structures in terms of toxicity and binding efficiency, as shown by other tools, such as ACFIS 2.0 [70] and FragPELE [71].

When comparing the designed drugs to other established anti-cancer drugs, it was found that the candidate drugs have low similarities to other known anti-cancer drugs. Figure 13 shows this (full results shown in Supplementary Materials Table S18), with the highest Tanimoto similarity being only 0.2340, which indicates a generally low degree of overlap in the structures. This suggests that although the candidate drugs are meant for a chaperone protein which is inherently receptive to many types of ligands, it is still specific enough to possibly be able to target clusterin alone without interfering with other drug-protein interactions. Despite the weak overlapping of the molecular fingerprints, some structural features are still conserved among the candidate drugs and established anti-cancer drugs; this can be seen in Supplementary Materials Figure S23.

A common point of similarity across clusters 0, 1, and 3 is their structural overlaps with small molecule inhibitors. Such drugs are responsible for protein binding, targeting key signaling pathways, modulation of immune cell activity, and interrupting the tumor microenvironment. Among nitrogen-containing anti-cancer drugs, one of the most common structures is that of pyridine. It was found that this substructure is involved in drugs that function as anti-tumor and anti-proliferation agents, cell-cycle regulators, and cytotoxic agents through challenging binding with their target proteins [72]. In addition, pyridazine is also implicated as a prominent contributor to the anti-cancer activity of certain drugs responsible for the inhibition of proteins involved in the onset and progression of cancer. Recent literature suggests that this substructure can act as either a core framework or as a warhead [73]. Both substructures were found to be part of the main fragment backbone of the peripheral structures, as shown in Figure 5a. In addition, the central structure is a fused pair of pyrrolidine rings. Although this substructure has been reported in some anti-cancer drugs and its derivatives can show anti-cancer activity, it is still undetermined if the pyrrolidine derivative found in the top 1 fragment, and consequently, the candidate drugs has any bearing on its potential anti-cancer activity. However, it should be noted that many anti-cancer drugs that feature pyrrolidine derivatives also prominently feature fused ring structures [74]. This suggests that there is a good probability that the candidate drugs can perform the same anti-cancer activities; however, much experimental testing is required to substantiate this hypothesis. Additionally, it was also noted that the added structures from the fragment growing were primarily responsible for making the candidates fit better on the clusterin structure, as evidenced by their 3D conformations and prominent polar groups that can serve as hydrogen bond acceptors and donors [75], as well as aromatic groups to fully utilize the aromatic interactions with the residues [76]. These observations are supported by the results shown in Figure 7.

When considering the properties of the top 1 final candidate drug precursor, it is important to note that the molecular dynamics simulation showed the ligand to be highly flexible within the binding pocket. The fact that CLU is a chaperone protein designed to accommodate all sorts of ligands means that it is unlikely to have a very firm binding site for a very specific type of ligand [46]. Although this runs counter to the principle of ligand-based design, flexible ligands do have potential uses that may prove useful to various treatment opportunities. This can be achieved through the functionalization of the peripheral sections, as shown in Figure 10f,g, of the top 1 final candidate drug precursor with a chemical group with proven effects. One such application is serving as a drug delivery system [77] for certain chemotherapy drugs such as gemcitabine, whilst simultaneously providing some degree of CLU suppression. Another potential route of application for flexible ligands is to serve as facilitators for certain protein-protein interactions, such as reported by Liu, B. et al. (2024), in which they suggested using small molecule ligands as potential facilitators for interactions with certain ubiquitin ligases, which directly degrade the protein [78]. This strategy also applies to any protein-protein interactions, especially in the case of chaperone proteins such as CLU as previously highlighted in this section [79]. Flexible ligands also have a role in multi-directed ligands whereby the non-specific structure may serve to benefit the drug due to being applicable to related proteins or even other proteins that have similar binding site characteristics [80]. Although the results of the molecular dynamics simulations show many potential scenarios of the bound top 1 final candidate drug precursor and the sCLU complex, it is apparent that there are still many conformational states that have not been covered. This can be seen in how Kruskal–Wallis [81] (Supplementary Materials Table S19) and Dunn’s Pairwise tests [82] (Supplementary Materials Table S20) show significant differences across all runs in RMSD, radius of gyration, and all energy terms from MMGBSA. We recommend more simulations in future work to properly ascertain the full range of behavior across many possible initial conditions. Another notable limitation is that MMGBSA does not account for explicit entropy and ligand dissolution. In this study, MMGBSA was primarily employed to compare the relative stability of the complexes, rather than for comprehensive free energy prediction. For future research, we recommend integrating wet-lab experiments to ascertain therapeutic effects, as these cannot be fully captured through in-silico predictions alone. The complete MMGBSA results are detailed in Supplementary Materials Table S21.

Although FBDD also has its shortcomings in terms of chemical space coverage, being biased and limited in some cases, initial fragment binding causing relatively low binding scores, which potentially filter out better drugs once grown, the presence of potential false negatives or false positives among the candidate drugs, synthesizability, and specificity [47], these results serve to illustrate the capability of the FBDD pipeline to produce biologically relevant results, with success stories in the form of vemurafenib [83], erdafitinib [84], pexidartinib [85], and venetoclax [86], as well as at least 40 different fragment-based candidate drugs for all types of conditions [87]. It is clear that this pipeline is capable of developing potential drugs at a much lower cost. This study provides insight into the capabilities of FBDD by identifying and characterizing the main ligand binding sites as well as potential allosteric sites. In addition, the candidate drugs in this study may be used as the basis for potential drugs, probes for biomarkers, and even delivery systems for other types of treatment. This highlights the flexibility of the method in producing compounds with a wide array of potential functions depending on the circumstance.

## 5. Conclusions

In conclusion, the study was able to discover potential drugs that can have an effect on the activity of sCLU, as indicated by the binding efficiency of the candidate drugs, as shown in Figure 6. These drugs would, however, require further optimization to address the issue of specificity and toxicity, as well as potentially further improving the binding efficiency of the candidate drugs. From the full database of 194 candidates, two were found to possess the proper ADME properties to ensure the reduction of toxicity as well as side-stepping any potentially harmful drug interactions. One of these showed favorable lipophobicity, which was deemed relevant to the subject of the study, which was to target sCLU. Molecular dynamics simulation of the top 1 final candidate drug precursor showed good potential for relatively stable binding when compared to the reference ligand NAG. Despite the stable and relatively strong binding affinity, the ligand was shown to be flexible within the binding site, which presents the opportunity to further functionalize the drug to impart unique functions, such as in drug delivery, multi-directed ligand binding, and targeting or facilitating protein-protein interactions. It was also found that the hotspot analysis conducted to discover the potential binding sites of sCLU was indeed able to identify these sites of interest. Both the potential main ligand binding sites, illustrated in Figure 3, and potential allosteric or protein-protein interaction sites, illustrated in Figure 13, were found using this approach. Further analysis of these sites would be required, however, to fully ascertain their function and behavior in the presence of certain stimuli. In addition, QSAR analysis was also conducted to determine the relationship of the chemical and structural descriptors to the binding efficiency from the docking predictions. It was determined that the overall complexity of the molecule contributed the most to the predictions made of the binding efficiencies of the model. In terms of accuracy, the model scores, shown in Table 5 and Figure 8b, indicate that the model shows acceptable predictions, with a test score of 0.63. The applicability domain, displayed in Figure 8c and Table 7, was also analyzed, wherein it was found that four distinct clusters of chemical space were covered by the model. Modifications to the model’s hyperparameters may result in a better model than the one presented in this study. In addition, testing the model’s applicability domain using new input compounds may also provide further insight into the robustness of the model. For future work, it is recommended to conduct molecular dynamics simulations to further describe the interactions between the other candidate drugs and sCLU. Additional recommendations are wet-lab experiments to capture behavior that cannot be ascertained in the limited window of molecular dynamics simulations.

## Figures and Tables

**Table 1:** Ten example fragments from the final database.

SMILES	GCA ^1^	SA ^2^	MW ^3^	Rot. Bonds ^4^	HBA ^5^	HBD ^6^	LogP
OCC(Br)(Br)Br	Active	61.5	282.76	0	1	1	1.82
CC1=CC=C(N=C1)C1=NC=C(C)C=C1	Active	83.66	184.24	1	2	0	2.76
Cl.CC(=O)C1=C(C)N=C(C)S1	Active	76.07	191.68	1	3	0	2.38
Cl.Cl.CNC(C#N)C1=CC=CN=C1	Active	90	220.1	2	3	1	1.71
N#CCC1=NC(=CS1)C1=CC=CO1	Active	79.27	190.23	2	4	0	2.47
CC1=CN2C=C(Br)C(C)=CC2=N1	Active	79.37	225.09	0	2	0	2.71
N#CC1(CCCCC1)N1CCCC1	Active	80.53	178.28	1	2	0	2.31
FC(F)(F)C1=CN=C(Br)C=C1	Active	69.38	226	0	1	0	2.86
N#CC1=NC(=CC=C1)C1=CC=CN1	Active	75.68	169.19	1	2	1	1.95
FC(F)(F)C1=CC=C(Br)N=C1	Active	69.38	226	0	1	0	2.86

^1^ General Cancer Activity, ^2^ Surface Area, ^3^ Molecular Weight, ^4^ Number of Rotatable Bonds, ^5^ Number of Hydrogen Bond Acceptors, ^6^ Number of Hydrogen Bond Donors.

**Table 2:** Binding clusters found in the docking experiment and their respective adjacent residues.

Color	Associated Residues	Population
gray	ARG167, ARG289	3
blue	GLU164, ASN165, HIS290, THR293, ASP157, ARG158, GLU335, PHE143, ASP160, ARG297, GLU287, TYR151, ASN291, SER161	399
red	SER210, PRO212, PHE195, ARG194, GLN201, GLU199	79
green	LEU211, ALA373, THR376	45
yellow	PHE150, LYS96, TYR151, SER148	12
purple	LEU375, GLY378	10
orange	GLU326, GLU87	2
cyan	LYS40, SER39, ASN43, TYR383	3
pink	SER356, ASP259, GLN255	2

**Table 3:** Top 10 fragments with the highest binding efficiency.

SMILES	Affinity (kcal/mol)
CC1=CC(=NN=C1C)N2CCC3C2CN(C3)C4=CC=CC=N4	−8.0
C=C1CC2CCC(C1)N2C(=O)C3=CC=C(C=C3)C(F)(F)F	−7.9
CC1=CN(N=C1)CC2CN(C2)C3=CC(=NC=C3)C(F)(F)F	−7.9
CC1=CC(=NC=N1)NC2CCCN(C2)C3=NC=C(C=C3)F	−7.9
CC1CCN(CC1)C2=NC=NC(=C2)N3C(=CC(=N3)C)C	−7.6
C1CCN(C1)C2CCN(CC2)C3=CC(=NC=C3)C(F)(F)F	−7.6
CC1=CC(=NC(=N1)N2CCCC(C2)C3=NN(C=C3)C)C	−7.4
CC1=CN(N=C1)C2CC3CCC(C2)N3C4=NC=C(C=C4)C#N	−7.4
CC1=CC(=NN=C1C)N2CCN(CC2)C3=CC=CC=N3	−7.4
C1CC2CCC1N2C3=NN4C(=NN=C4C(F)(F)F)C=C3	−7.4

**Table 4:** The top resulting candidate drugs with the highest docking efficiency with the predicted toxicities from T.E.S.T. structures illustrated using SMILES to Structure [53].

Structure	Affinity (kcal/mol)	Bioconcentration Factor	*D. magna* LC50 (mg/L)	Fathead Minnow LC50 (mg/L)	Oral Rat LD50 (mg/kg)	*T. pyriformis* IGC50 (mg/L)
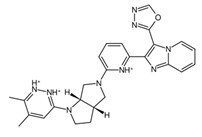	−10.4	N/A	0.8	0.04	1445.04	8.57
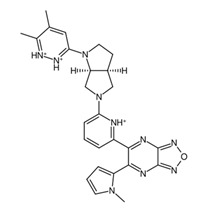	−10	22.77	7.27	0.05	593.3	8.84
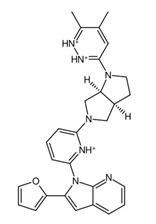	−9.8	9.82	19.39	0.01	80.34	5
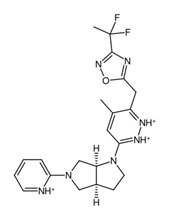	−9.8	8.82	0.65	0.19	95.57	3.12
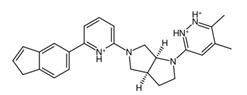	−9.8	N/A	3.48	0.01	1182.57	8

N/A: Not applicable.

**Table 5:** Top 5 highest-scoring ANN hidden layer architectures according to test scores.

Hidden Layer Sizes	Train Score	Test Score	Cross-Val Mean	Test RMSE	Normalized RMSE (%)
(20, 20, 130)	0.7	0.63	0.56	0.63	12.78
(10, 10, 20)	0.67	0.62	0.51	0.63	12.92
(30, 10, 20)	0.68	0.61	0.52	0.65	13.18
(40, 10, 30)	0.69	0.61	0.3	0.65	13.18
(10, 10, 40)	0.68	0.61	0.54	0.65	13.23

**Table 6:** QSAR model descriptor importance.

Descriptor	Importance	Variability	Short Description of the Descriptors
MaxAbsEStateIndex	0.03	±0.02178	Maximum EState index of the molecule
FpDensityMorgan3	0.01	±0.006007	Morgan fingerprint within a radius of 3 angstroms
BCUT2D_MWHI	0.06	±0.01858	Highest eigenvalue-weight using atomic masses
AvgIpc	0.42	±0.04927	Expression of molecular complexity
BalabanJ	0.14	±0.01229	Balaban’s J value, which states degree of branching
SlogP_VSA3	0.04	±0.01095	MOE logP VSA Descriptor 3 in a distance of −0.20 ≤ x < 0.00
NumRotatableBonds	0.04	±0.008200	The number of rotatable bonds
fr_Al_COO	0.02	±0.01681	Amount of aliphatic carboxylic acids
fr_Ar_OH	0	±0.0000	Amount of aliphatic hydroxyl groups
fr_alkyl_halide	0.02	±0.0009489	Amount of alkyl halides
fr_aryl_methyl	0	±0.02298	Amount of aryl methyl sites for hydroxylation
fr_lactam	0.08	±0.04856	Amount of beta lactams
fr_piperzine	0.04	±0.01956	Amount of piperzine rings

**Table 7:** Distinguishing features of each cluster found in the QSAR model’s applicability domain.

Population	Binding Affinity	Surface Area	Volume	Number of H-Bond Acceptors	Number of H-Bond Donors	Molecular Weight	Cluster
72	−8.425 ± 0.807	92.258 ± 17.303	435.225 ± 29.02	4.972 ± 1.061	0.556 ± 0.603	435.626 ± 29.134	0
60	−8.247 ± 0.577	86.809 ± 15.971	435.749 ± 30.593	4.85 ± 1.071	0.433 ± 0.563	436.091 ± 30.645	1
31	−6.481 ± 1.114	55.531 ± 24.922	276.887 ± 66.822	2.968 ± 1.329	1.29 ± 0.864	277.208 ± 66.975	2
31	−8.135 ± 0.632	86.413 ± 13.134	430.012 ± 28.115	4.484 ± 0.769	0.742 ± 0.815	430.324 ± 28.15	3

## Data Availability

The original contributions presented in this study are included in the article/Supplementary Materials. Further inquiries can be directed at the corresponding author.

## Supplementary Materials

The following supporting information can be downloaded at: https://www.mdpi.com/article/10.3390/biology14060639/s1, Table S1: Complete final fragment database; Table S2: Raw results from FTMap; Table S3: Full fragment docking experiment results; Table S4: Complete docking scores and toxicities of candidate drugs; Table S5: Hidden layer sizes scores; Table S6: ADMET results of candidate drugs with affinities greater than parent fragment; Table S7: Initial conditions of all molecular dynamics runs; Table S8: Complete data for RMSD of the top 1 final candidate drug precursor; Table S9: Complete data for RMSD of the reference ligand; Table S10: ADF test results for RMSD and radius of gyration for both ligands; Table S11: Complete data for radius of gyration of the top 1 final candidate drug precursor; Table S12: Complete data for radius of gyration of the reference ligand; Table S13: Summary statistics for RMSD and radius of gyration for both ligands across all runs; Table S14: ADF results for the MMGBSA energy terms for all runs of both ligands; Table S15: Summary statistics of the MMGBSA energy terms for all runs of both ligands; Table S16: Docking to clusterin results of known anti-cancer drugs; Table S17: Synthesizability results of all candidate drugs; Table S18: Tabular form of similarity analysis; Table S19: Kruskal–Wallis test results for all runs and all quantities; Table S20: Dunn’s Pairwise tests results for all runs and all quantities; Table S21: Complete MMGBSA results. Figure S1: Cluster members at each 20th percentile in terms of binding affinity; Figure S2: RMSD cumulative average plot for the top 1 final candidate drug precursor; Figure S3: RMSD cumulative average plot for the reference ligand; Figure S4: RMSD block averaging analysis plot for the top 1 final candidate drug precursor, block size = 50; Figure S5: RMSD moving window analysis plot for the top 1 final candidate drug precursor, window size = 100; Figure S6: RMSD block averaging analysis plot for the reference ligand, block size = 50; Figure S7: RMSD moving window analysis plot for the reference ligand, window size = 100; Figure S8: Radius of gyration cumulative average plot for the top 1 final candidate drug precursor; Figure S9: Radius of gyration cumulative average plot for the reference ligand; Figure S10: Radius of gyration block averaging plot for the top 1 final candidate drug precursor, block size = 50; Figure S11: Radius of gyration block averaging plot for the reference ligand, block size = 50; Figure S12: Radius of gyration moving window plot for the top 1 final candidate drug precursor, window size = 100; Figure S13: Radius of gyration moving window plot for the reference ligand, window size = 100; Figure S14: Illustrated poses for the remaining 3 runs at 20 ns, 40 ns, 60 ns, 80 ns, and 100 ns of the sCLU–Top 1 Final Candidate Drug Precursor complex; Figure S15: Additional plots of the remaining MMGBSA terms for the top 1 final candidate drug precursor; Figure S16: Additional plots of the remaining MMGBSA terms for the reference ligand; Figure S17: Additional cumulative average plots of the remaining MMGBSA terms for the top 1 final candidate drug precursor; Figure S18: Additional cumulative average plots of the remaining MMGBSA terms for the reference ligand; Figure S19: Block average analysis of the MMGBSA terms of the top 1 final candidate drug precursor, block size = 5 ns; Figure S20: Block average analysis of the MMGBSA terms of the reference ligand, block size = 5 ns; Figure S21: Moving window analysis of the MMGBSA terms of the top 1 final candidate drug precursor, window size = 50; Figure S22: Moving window analysis of the MMGBSA terms of the reference ligand, window size = 50; Figure S23: Top 5 known anti-cancer drugs with the highest similarities in each cluster.

## Abbreviations

The following abbreviations are used in this manuscript:

Fragment-based drug discovery	FBDD
Clusterin	CLU
Augmented Dickey-Fuller	ADF
Nuclear Clusterin	nCLU
Secretory Clusterin	sCLU
Qualitative Structure–Activity Relationship	QSAR
Principal Component Analysis	PCA
t-Distributed Stochastic Neighbor Embedding	t-SNE
Root Mean Square Error	RMSE
Root Mean Square Deviation	RMSD
Root Mean Square Fluctuation	RMSF
Molecular Mechanics with Generalized Born and Surface-area Solvation	MMGBSA
