# Supplementary figures and images for: Overcoming Clusterin-Induced Chemoresistance in Cancer: A Computational Study Using a Fragment-Based Drug Discovery Approach
Source: Biology (Basel). 2025 May 30;14(6):639. doi: 10.3390/biology14060639 (PMC12189888; doi:10.3390/biology14060639)

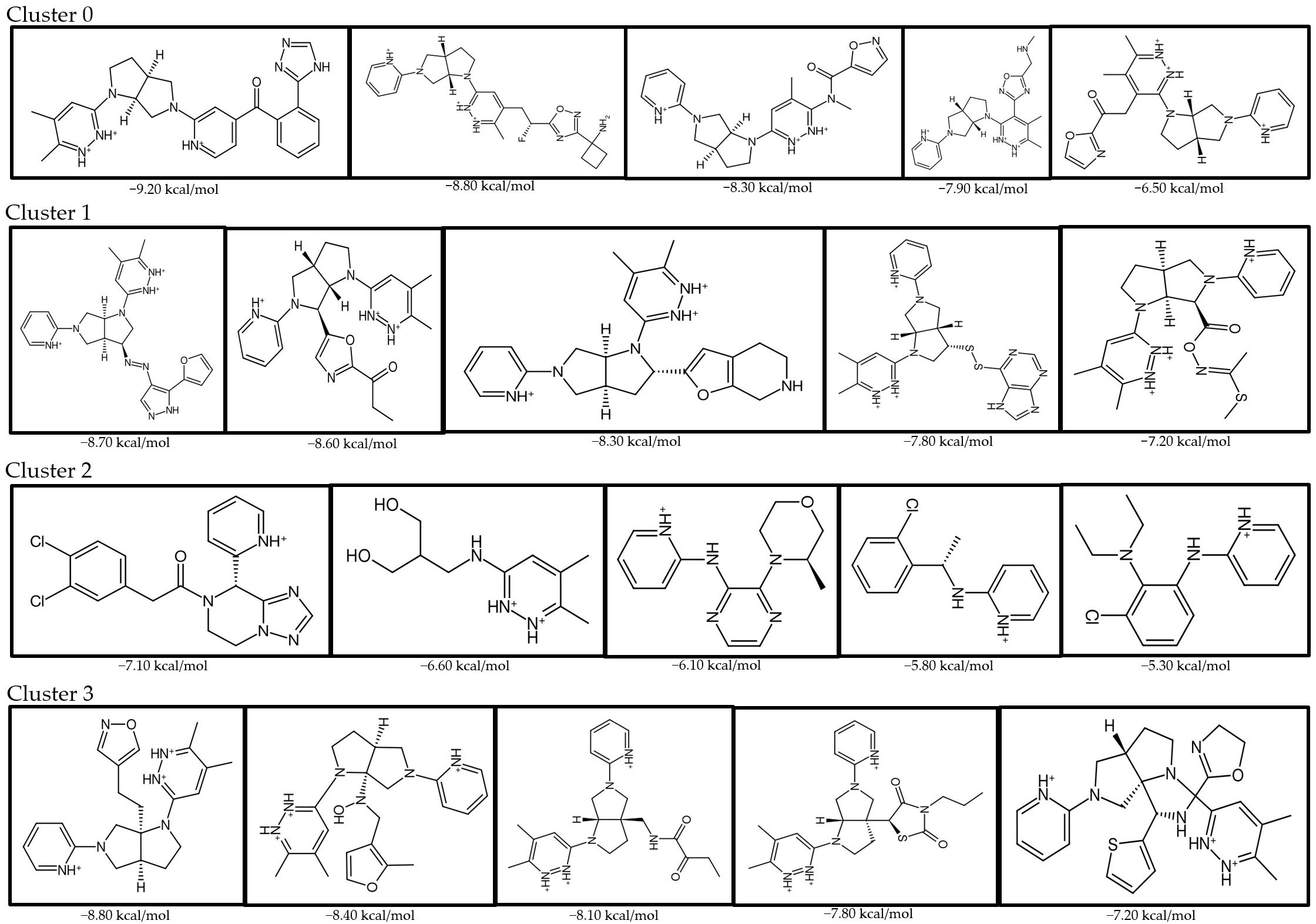

Supplement: Supplementary file 1 [file biology-14-00639-s001.zip › Supplemental Figures/Figure S1. Cluster members at each 20th percentile in terms of binding affinity.jpg]

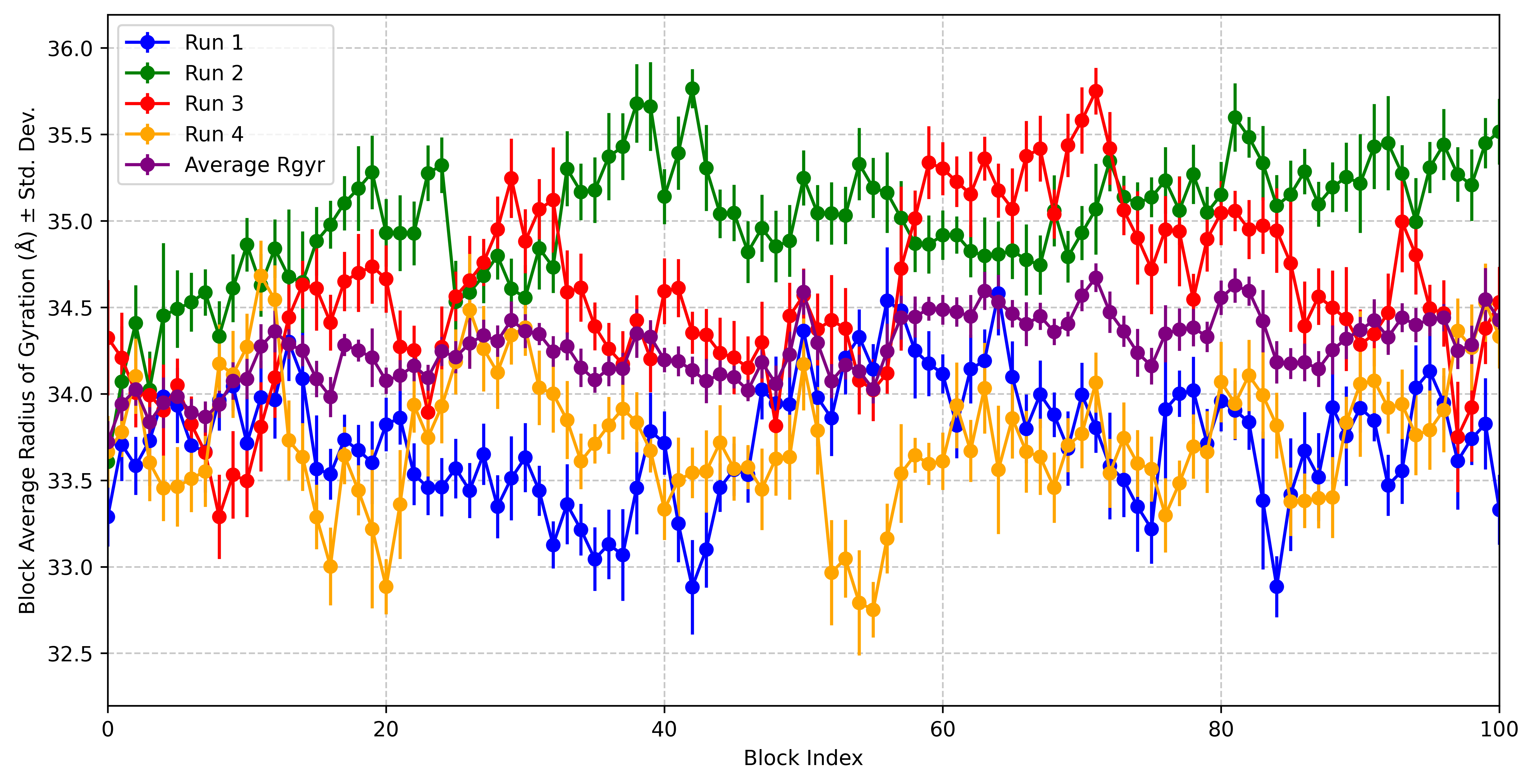

Supplement: Supplementary file 1 [file biology-14-00639-s001.zip › Supplemental Figures/Figure S10. Radius of gyration block averaging plot for the top 1 final candidate drug precursor, block size = 50.png]

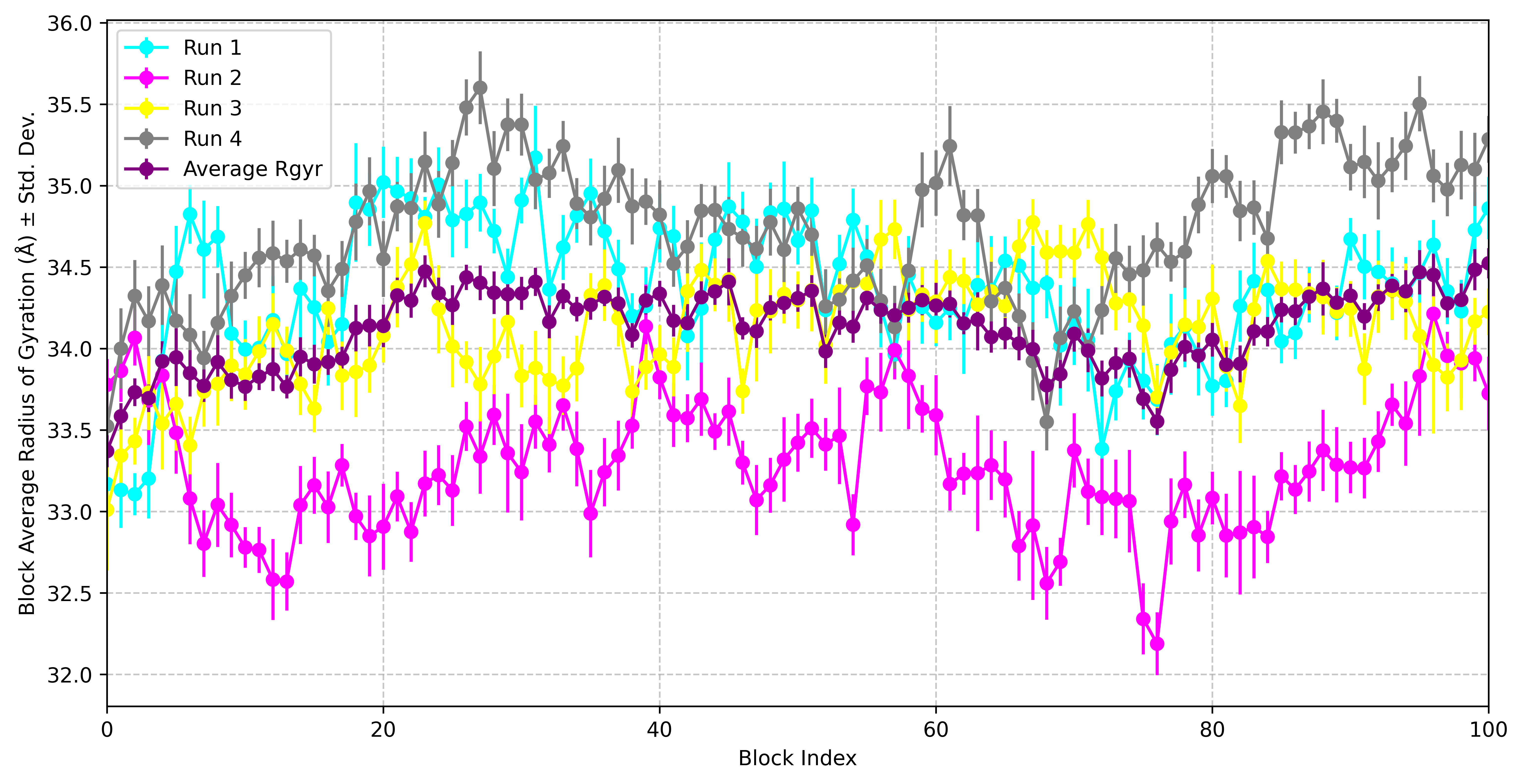

Supplement: Supplementary file 1 [file biology-14-00639-s001.zip › Supplemental Figures/Figure S11. Radius of gyration block averaging plot for the reference ligand, block size = 50.png]

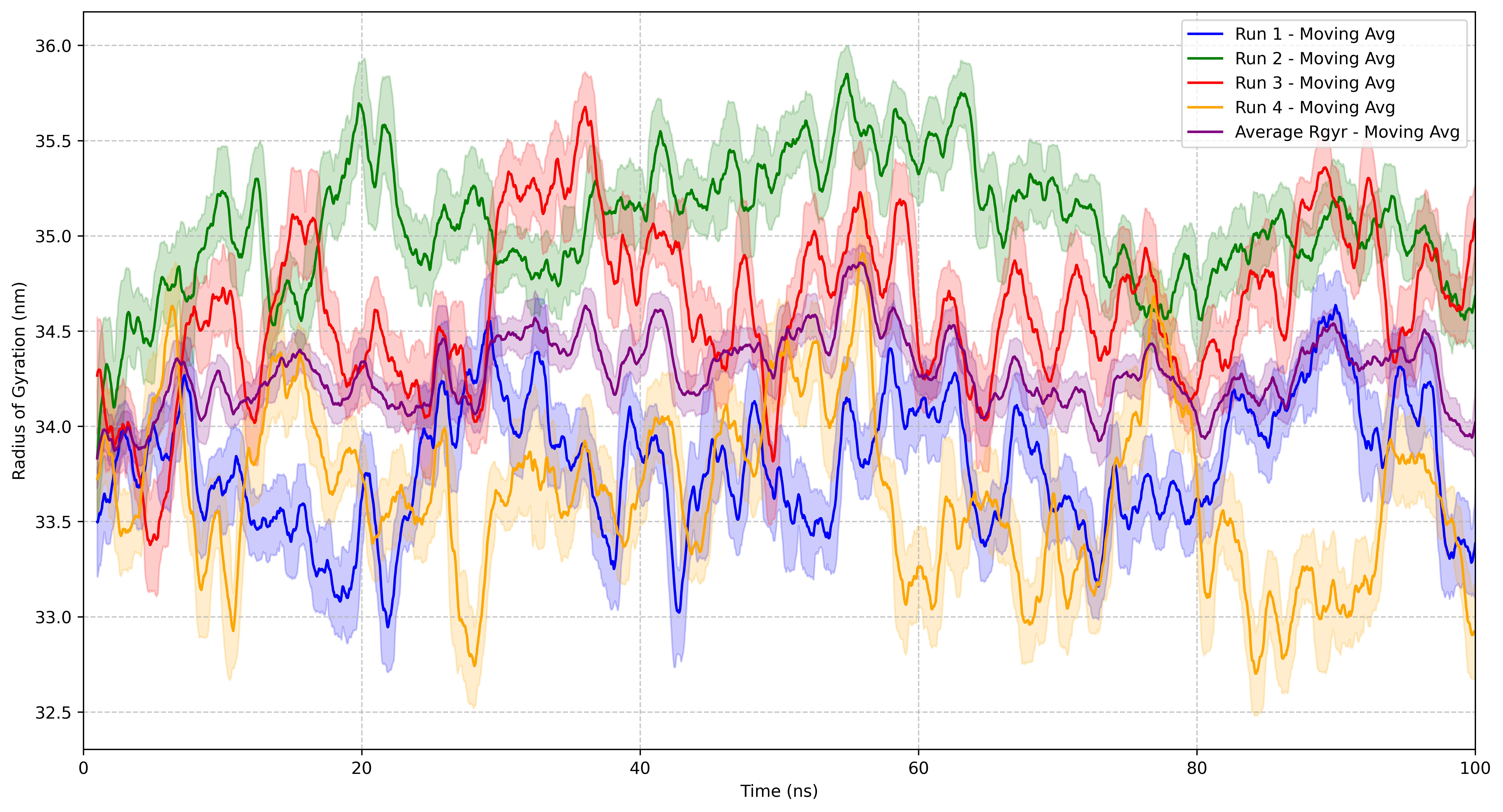

Supplement: Supplementary file 1 [file biology-14-00639-s001.zip › Supplemental Figures/Figure S12. Radius of gyration moving window plot for the top 1 final candidate drug precursor, window size = 100.png]

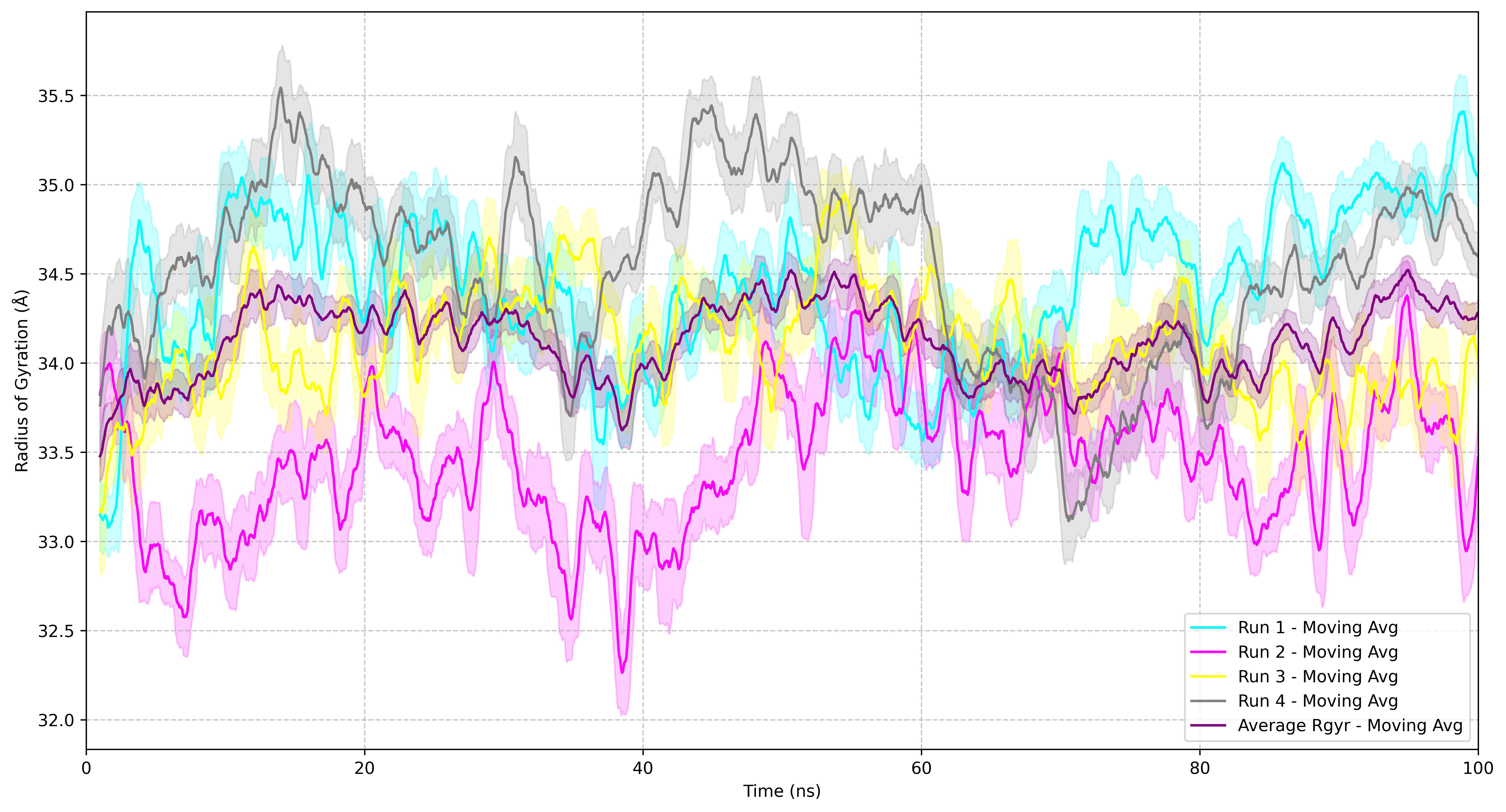

Supplement: Supplementary file 1 [file biology-14-00639-s001.zip › Supplemental Figures/Figure S13. Radius of gyration moving window plot for the reference ligand, window size = 100.png]

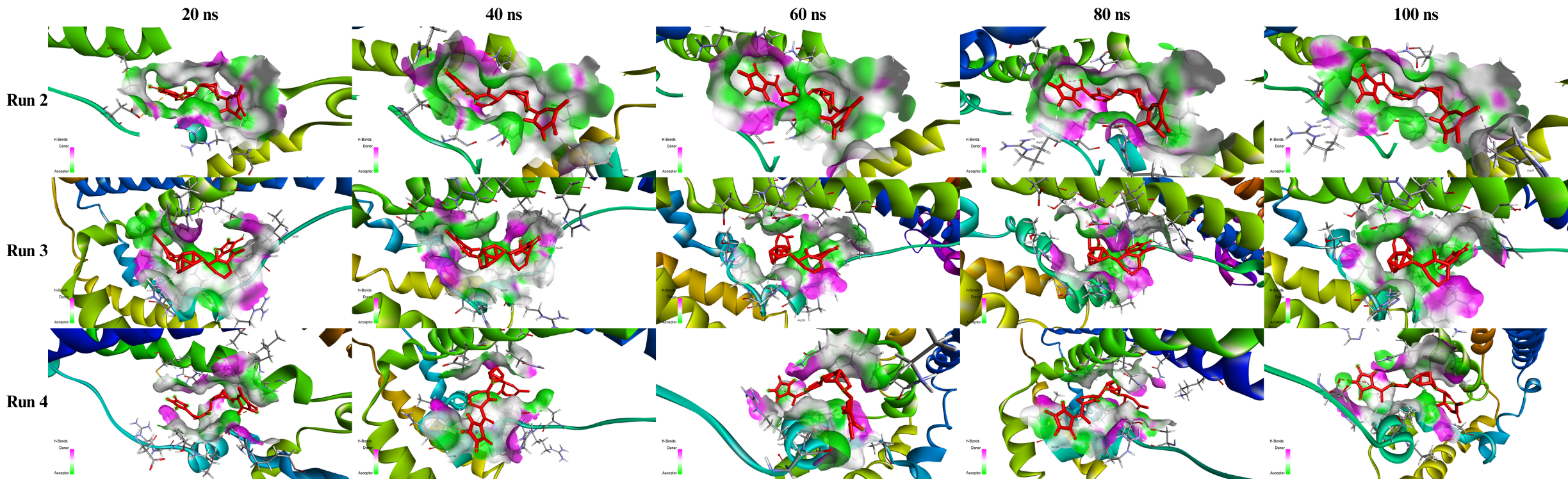

Supplement: Supplementary file 1 [file biology-14-00639-s001.zip › Supplemental Figures/Figure S14. Illustrated poses for the remaining 3 runs at 20ns, 40ns, 60ns, 80ns, and 100ns of the sCLU-Top 1 Final Candidate Drug Precursor complex.png]

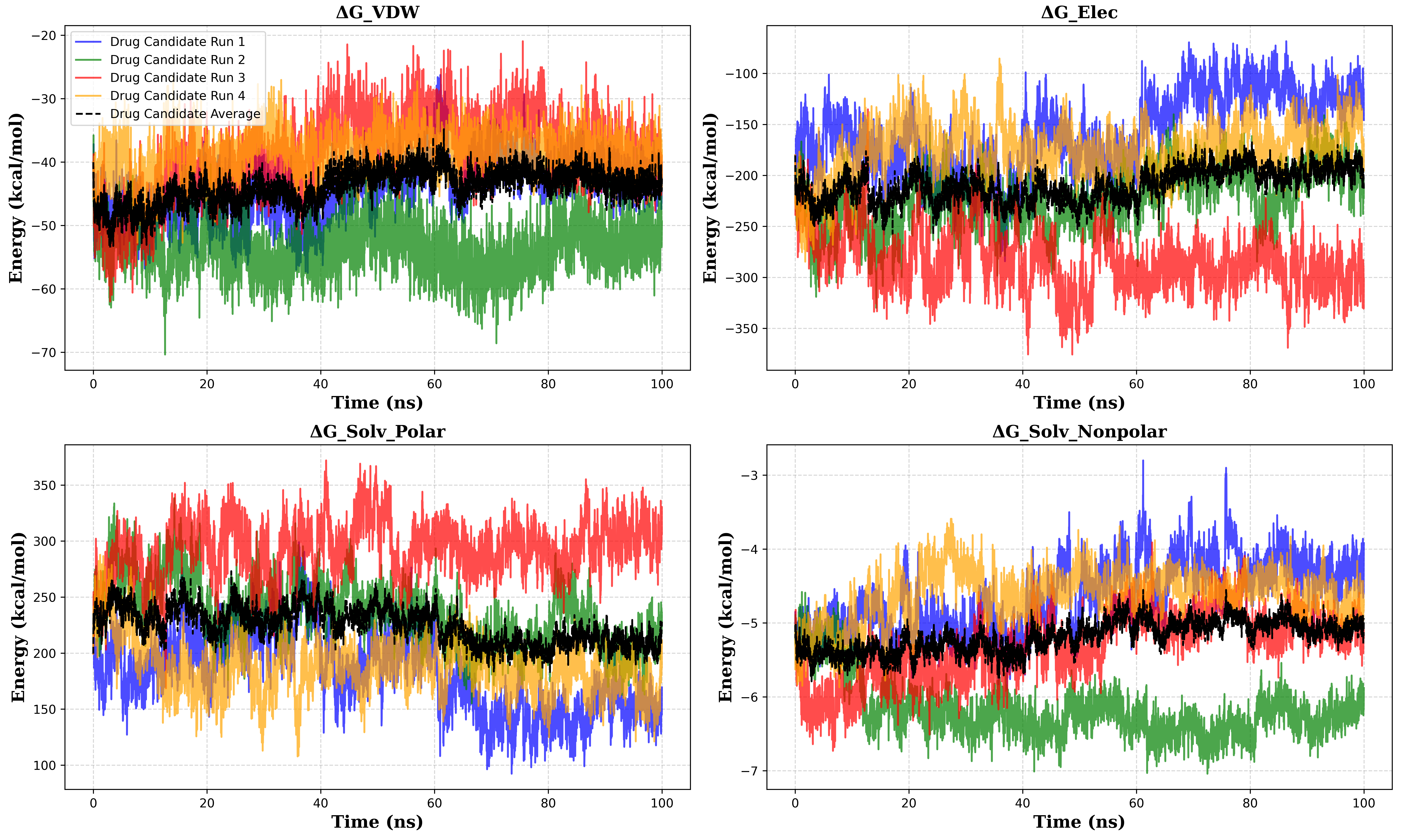

Supplement: Supplementary file 1 [file biology-14-00639-s001.zip › Supplemental Figures/Figure S15. Additional plots of the remaining MMGBSA terms for the top 1 final candidate drug precursor.png]

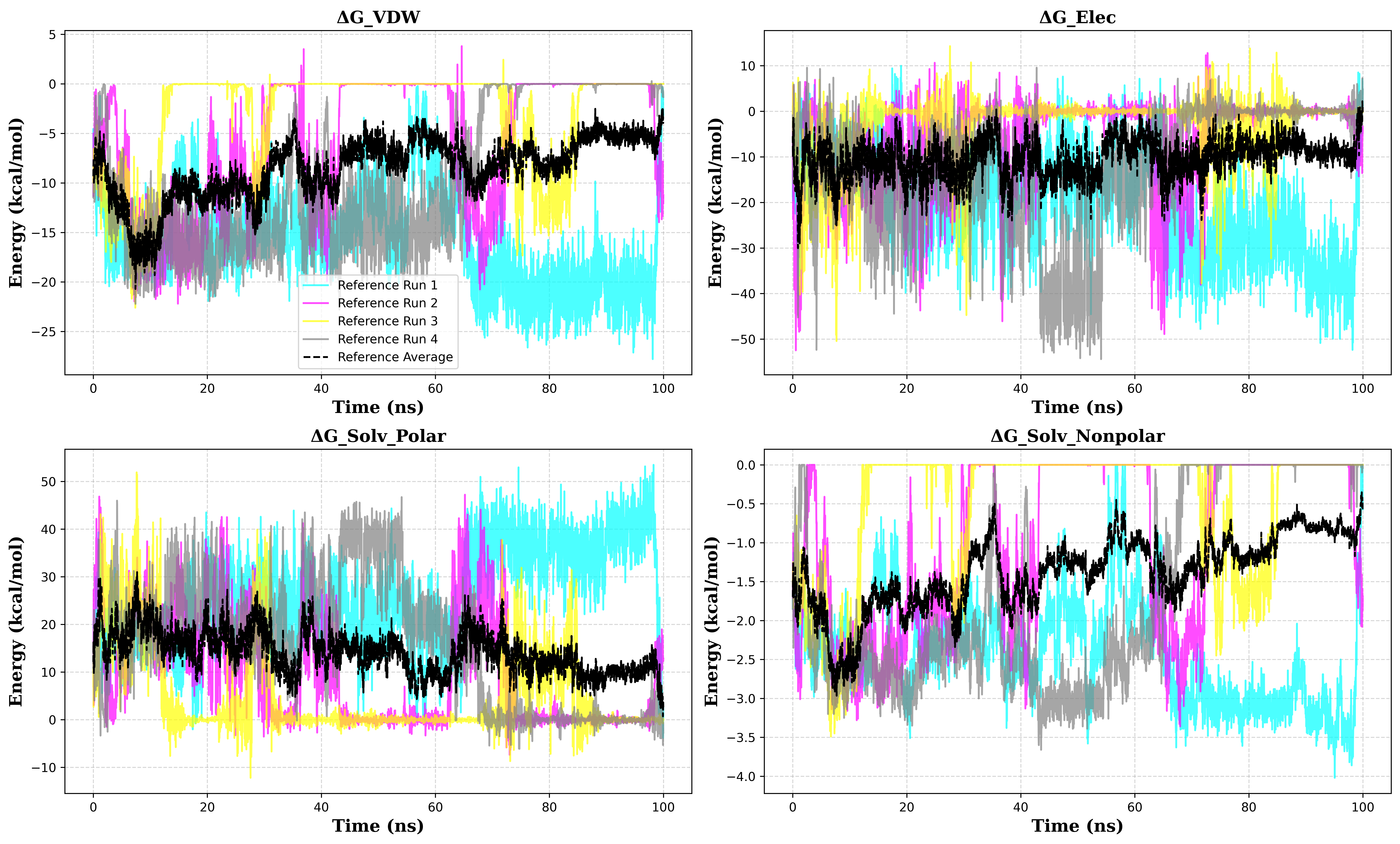

Supplement: Supplementary file 1 [file biology-14-00639-s001.zip › Supplemental Figures/Figure S16. Additional plots of the remaining MMGBSA terms for the reference ligand.png]

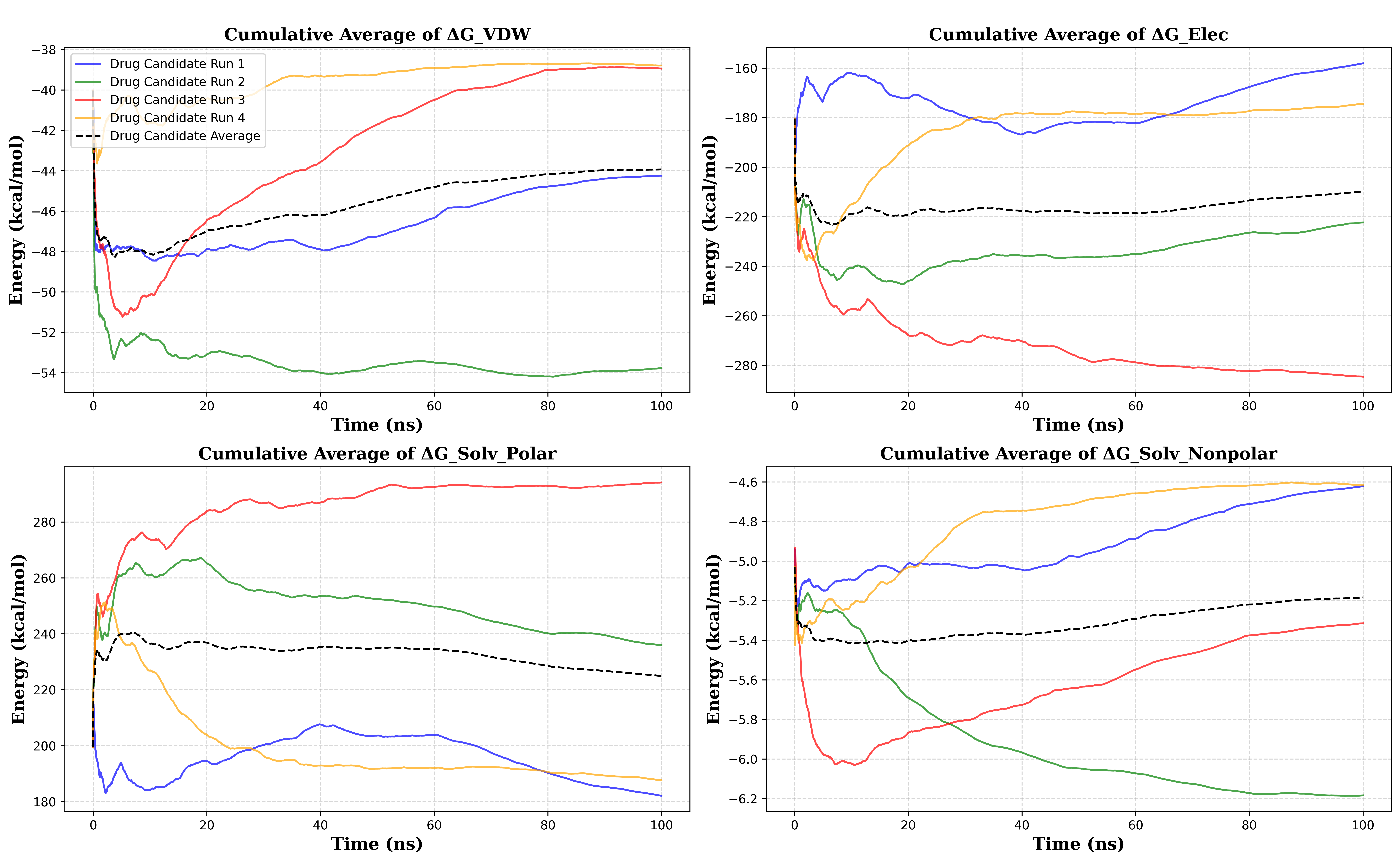

Supplement: Supplementary file 1 [file biology-14-00639-s001.zip › Supplemental Figures/Figure S17. Additional cumulative average plots of the remaining MMGBSA terms for the top 1 final candidate drug precursor.png]

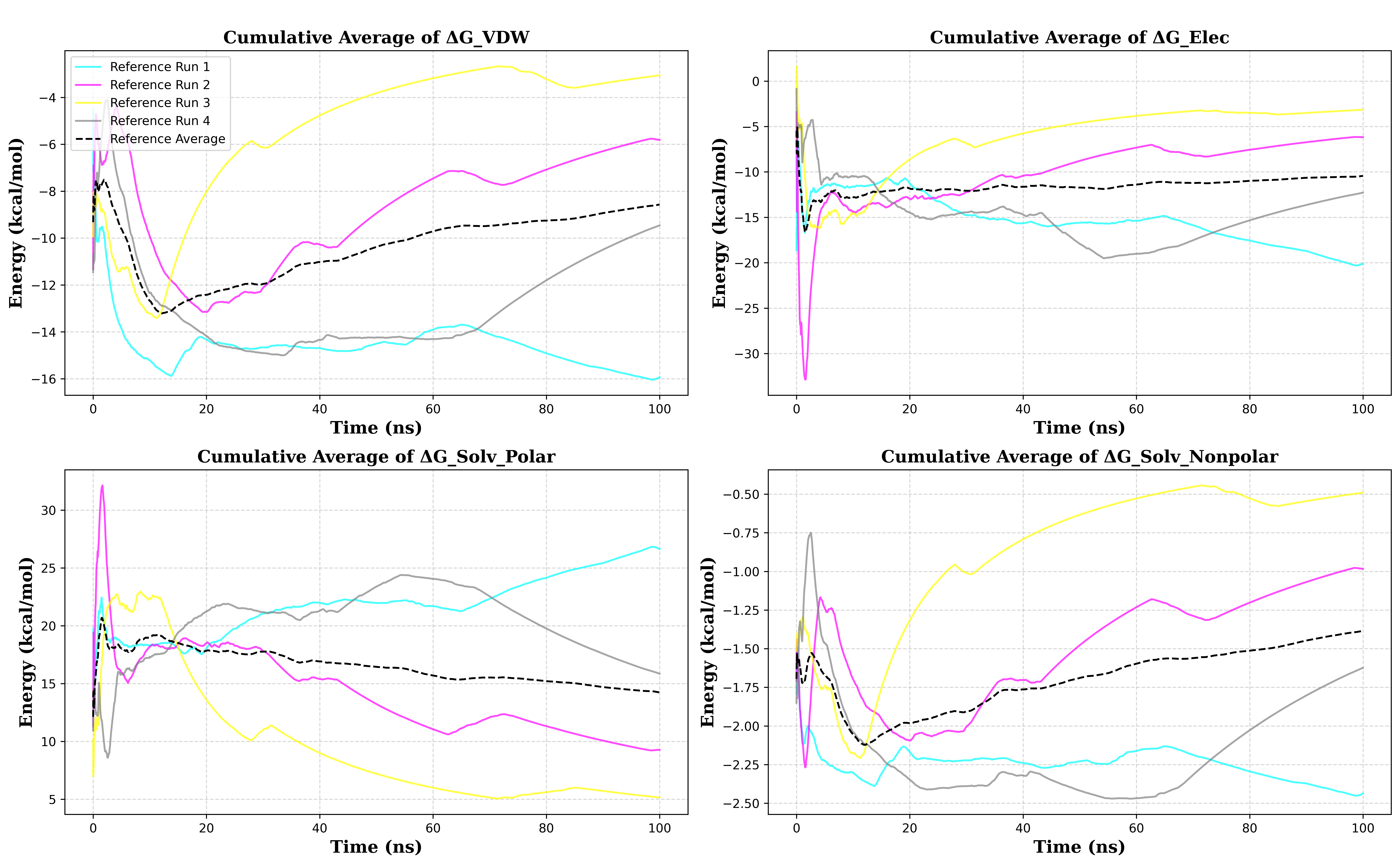

Supplement: Supplementary file 1 [file biology-14-00639-s001.zip › Supplemental Figures/Figure S18. Additional cumulative average plots of the remaining MMGBSA terms for the reference ligand.png]

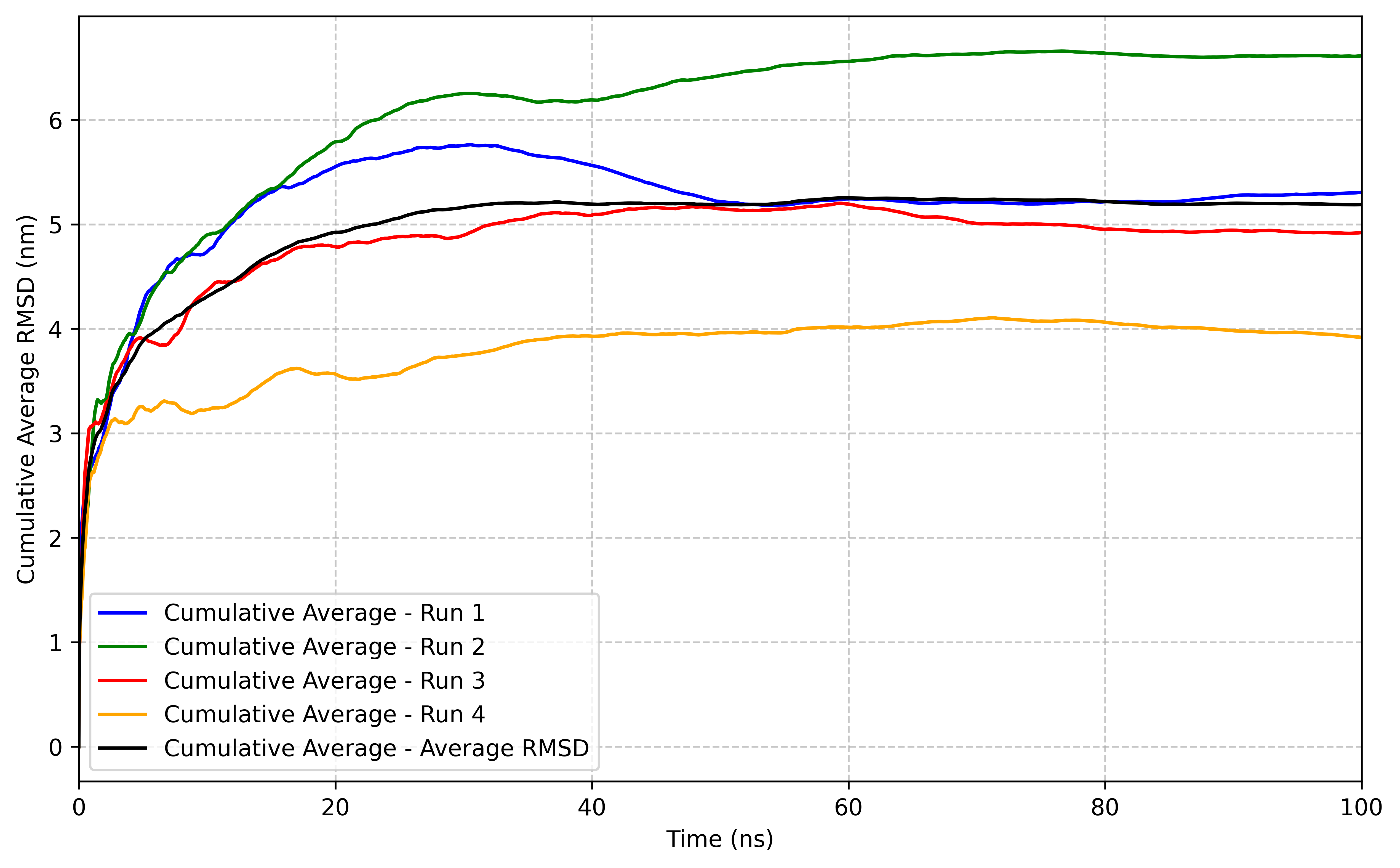

Supplement: Supplementary file 1 [file biology-14-00639-s001.zip › Supplemental Figures/Figure S2. RMSD cumulative average plot for the top 1 final candidate drug precursor.png]

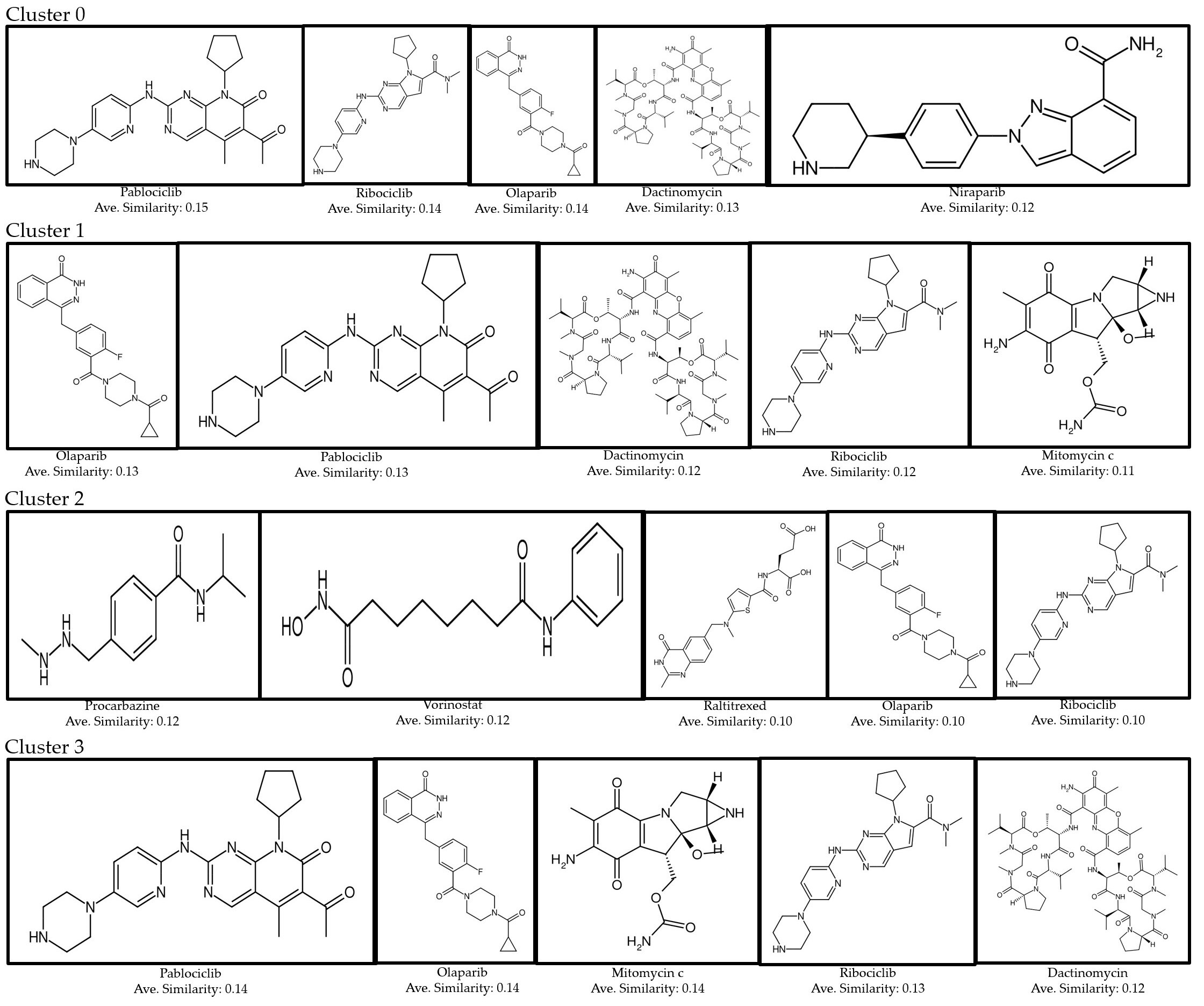

Supplement: Supplementary file 1 [file biology-14-00639-s001.zip › Supplemental Figures/Figure S23. Top 5 known anti-cancer drugs with the highest similarities in each cluster.jpg]

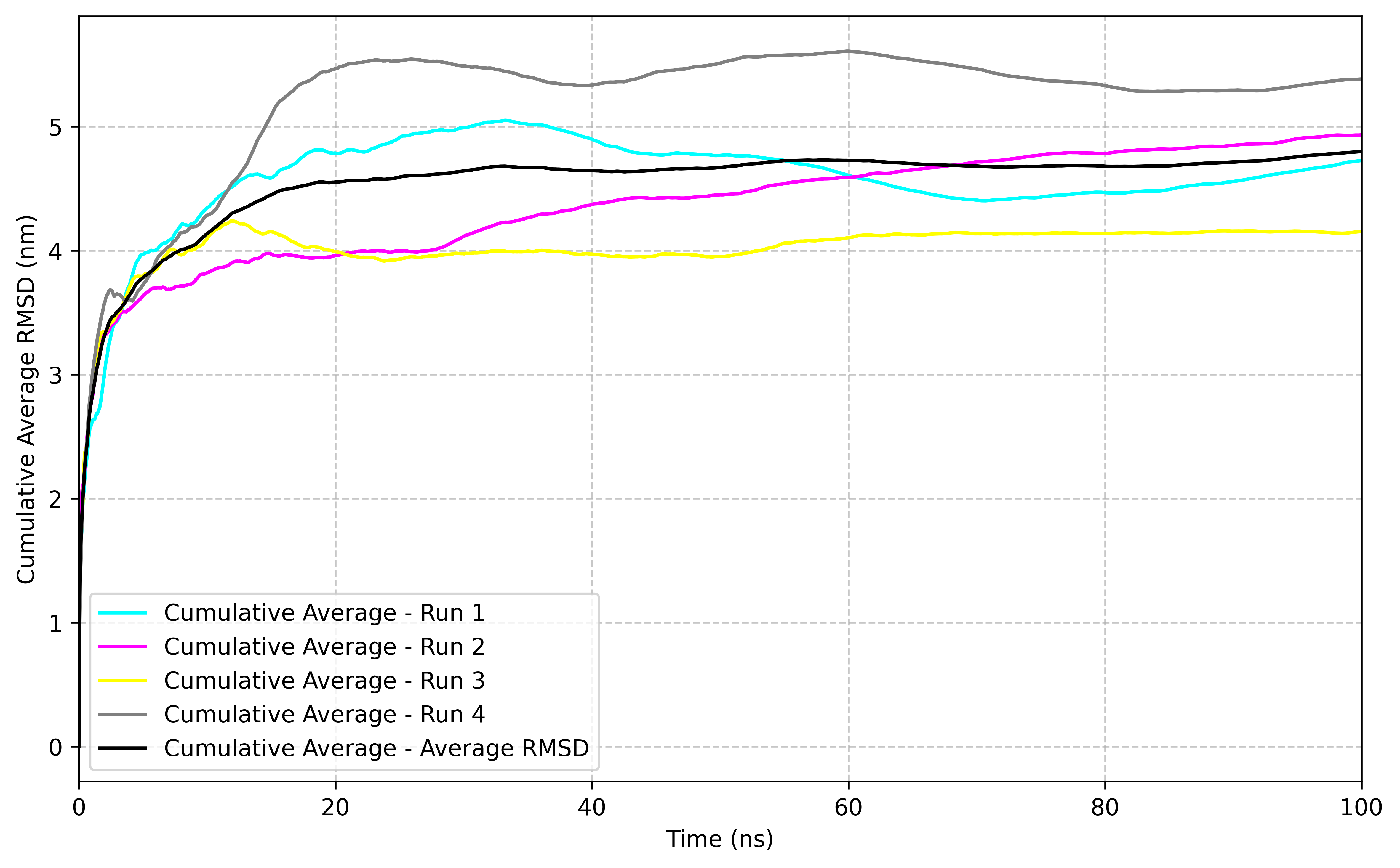

Supplement: Supplementary file 1 [file biology-14-00639-s001.zip › Supplemental Figures/Figure S3. RMSD cumulative average plot for the reference ligand.png]

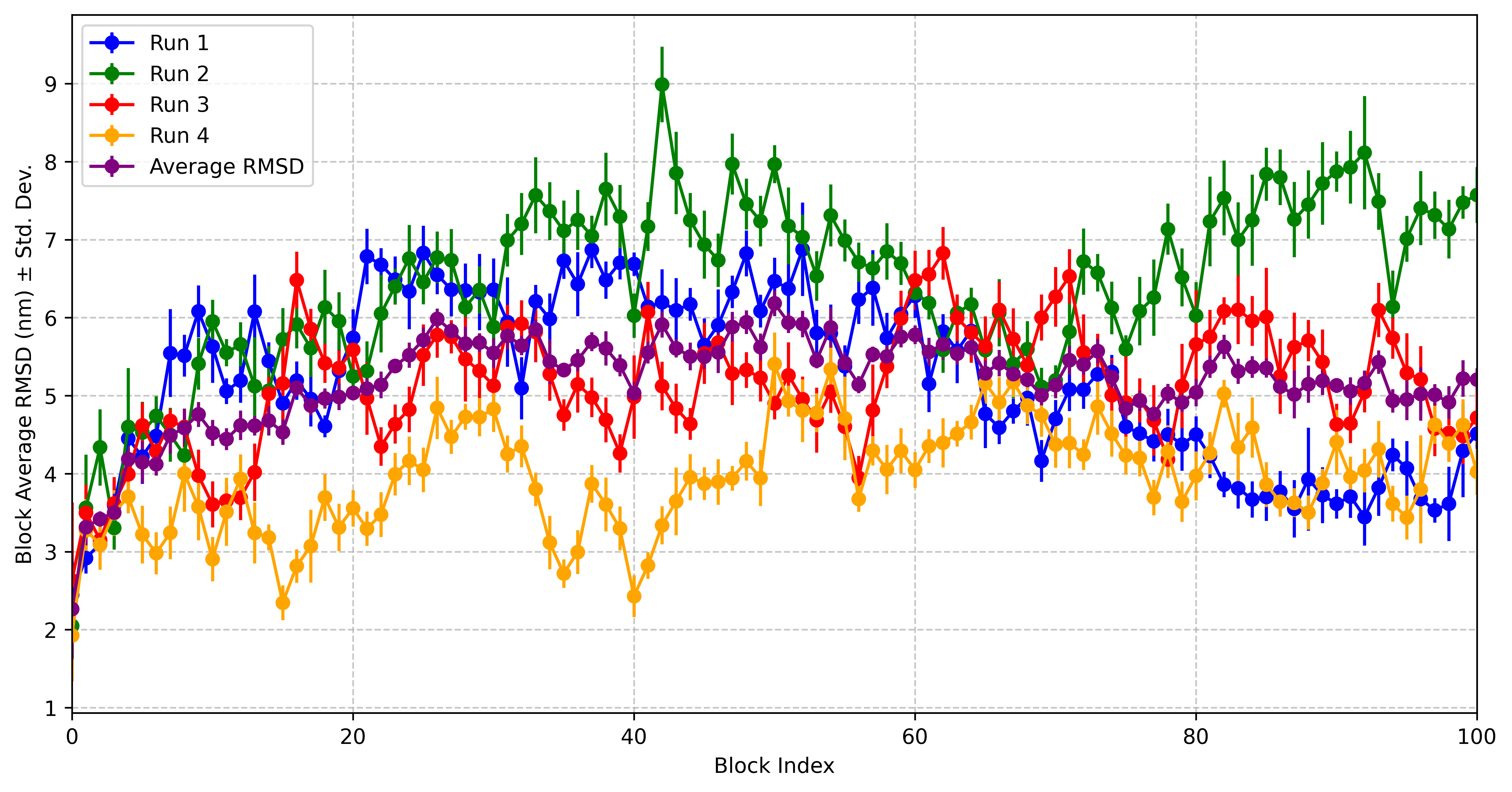

Supplement: Supplementary file 1 [file biology-14-00639-s001.zip › Supplemental Figures/Figure S4. RMSD block averaging analysis plot for the top 1 final candidate drug precursor, block size = 50.png]

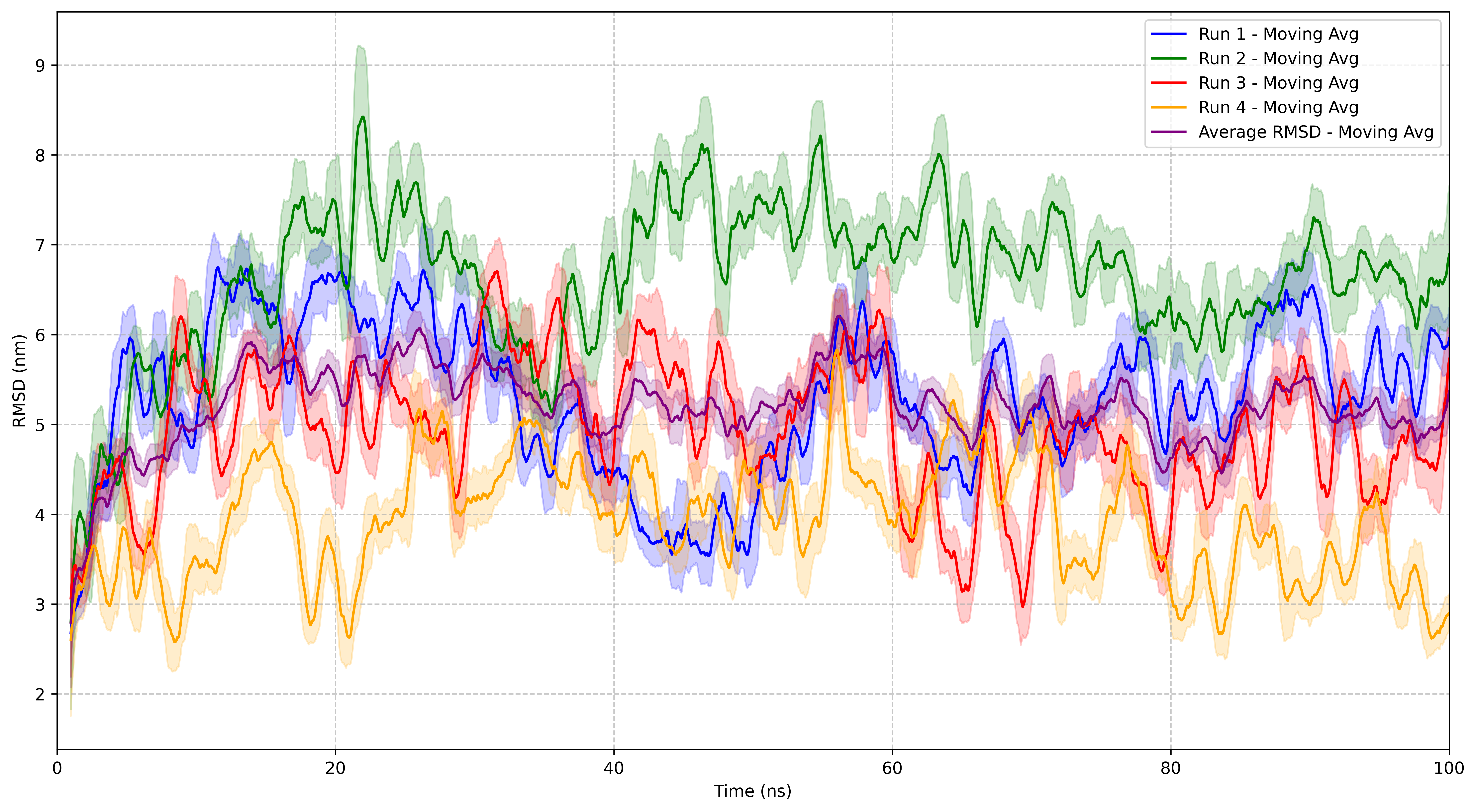

Supplement: Supplementary file 1 [file biology-14-00639-s001.zip › Supplemental Figures/Figure S5. RMSD moving window analysis plot for the top 1 final candidate drug precursor, window size = 100.png]

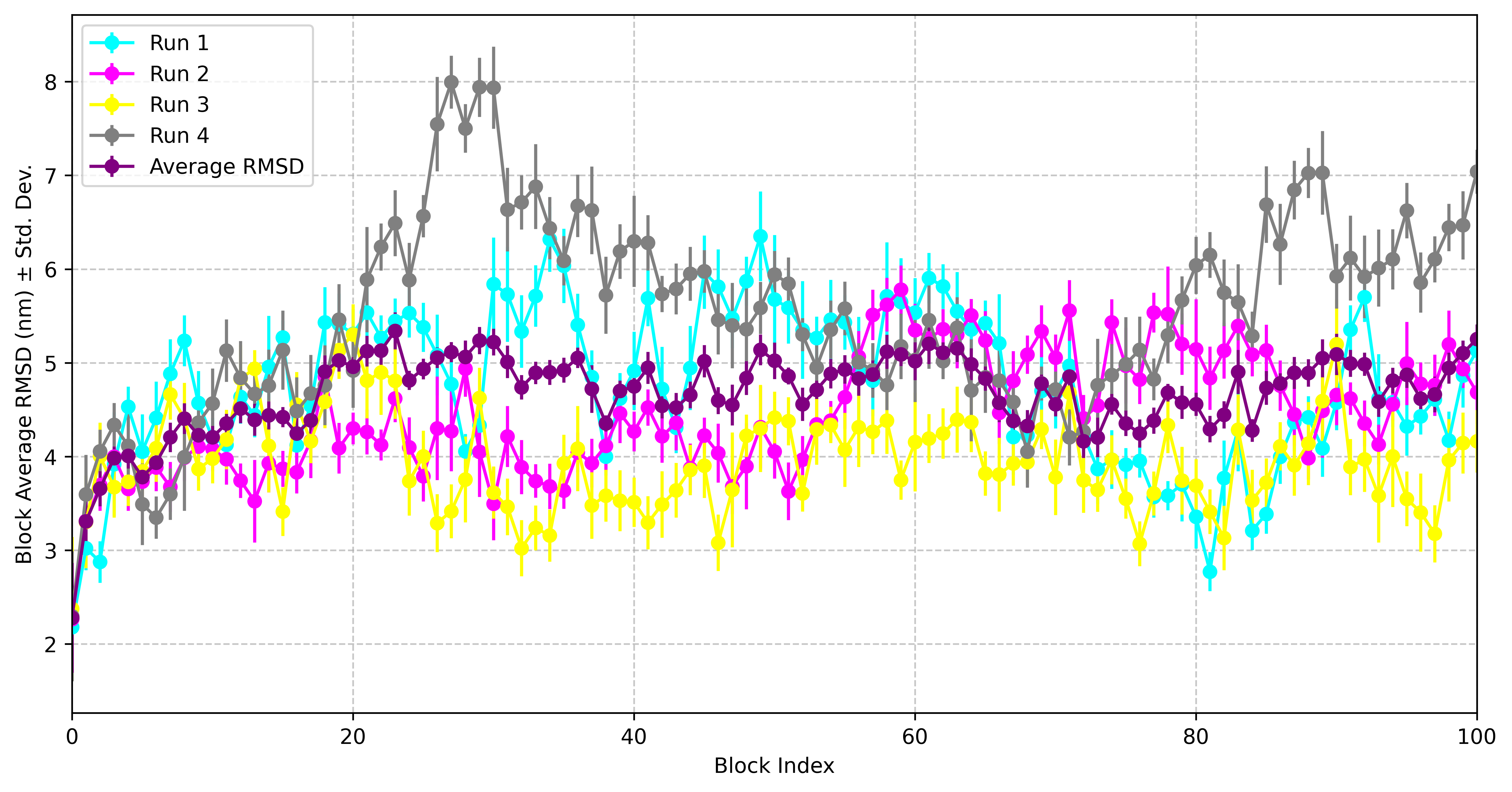

Supplement: Supplementary file 1 [file biology-14-00639-s001.zip › Supplemental Figures/Figure S6. RMSD block averaging analysis plot for the reference ligand, block size = 50.png]

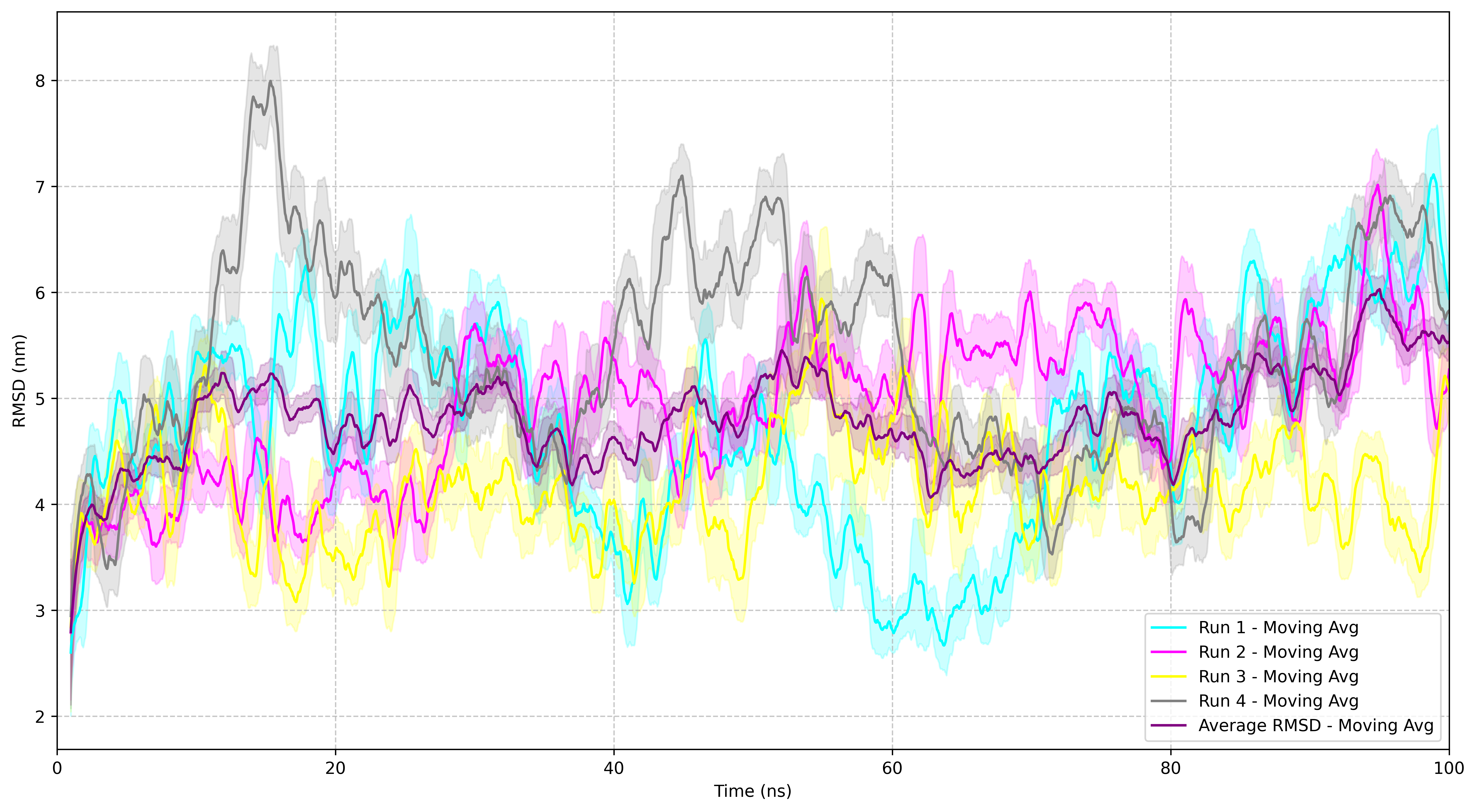

Supplement: Supplementary file 1 [file biology-14-00639-s001.zip › Supplemental Figures/Figure S7. RMSD moving window analysis plot for the reference ligand, window size = 100.png]

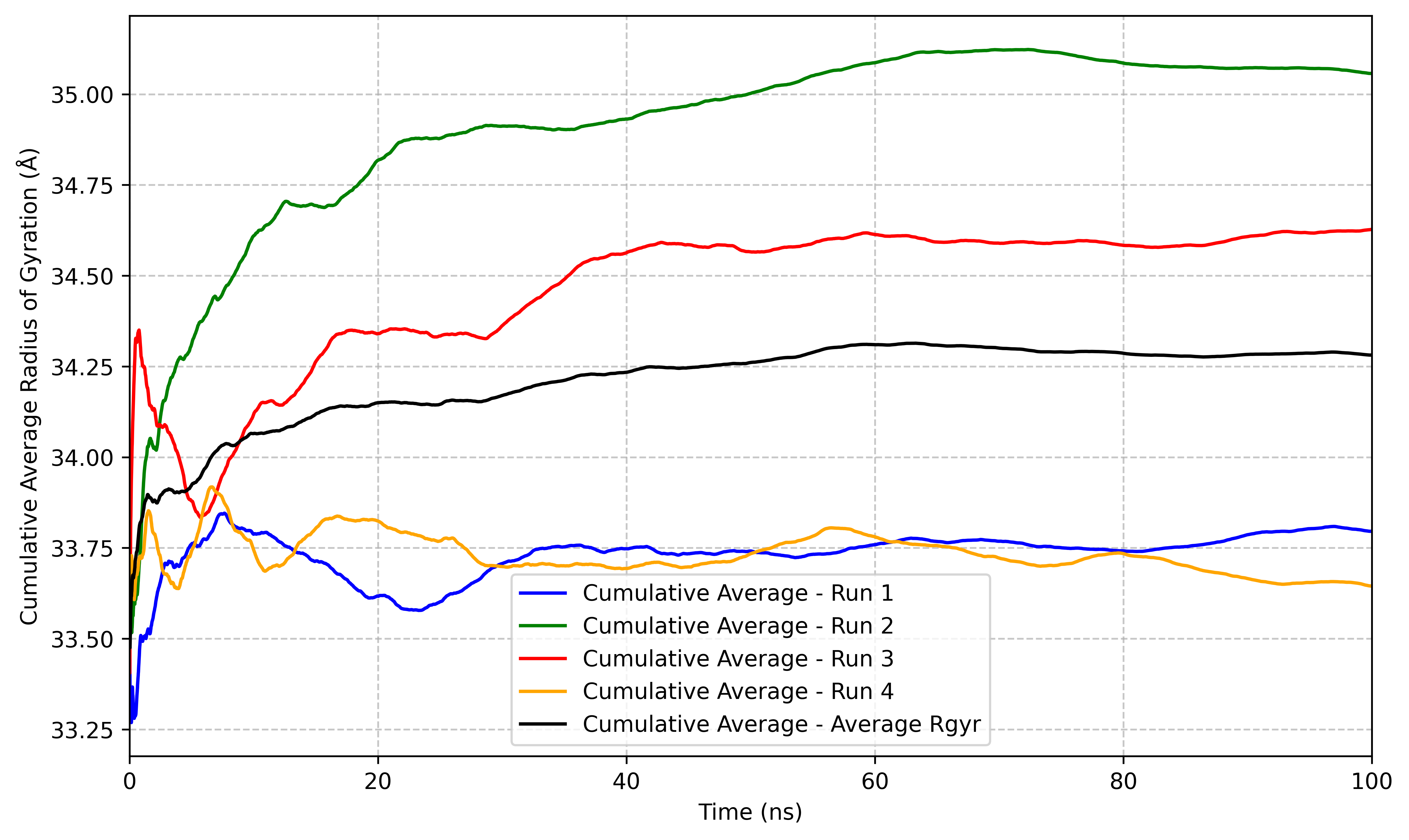

Supplement: Supplementary file 1 [file biology-14-00639-s001.zip › Supplemental Figures/Figure S8. Radius of gyration cumulative average plot for the top 1 final candidate drug precursor.png]

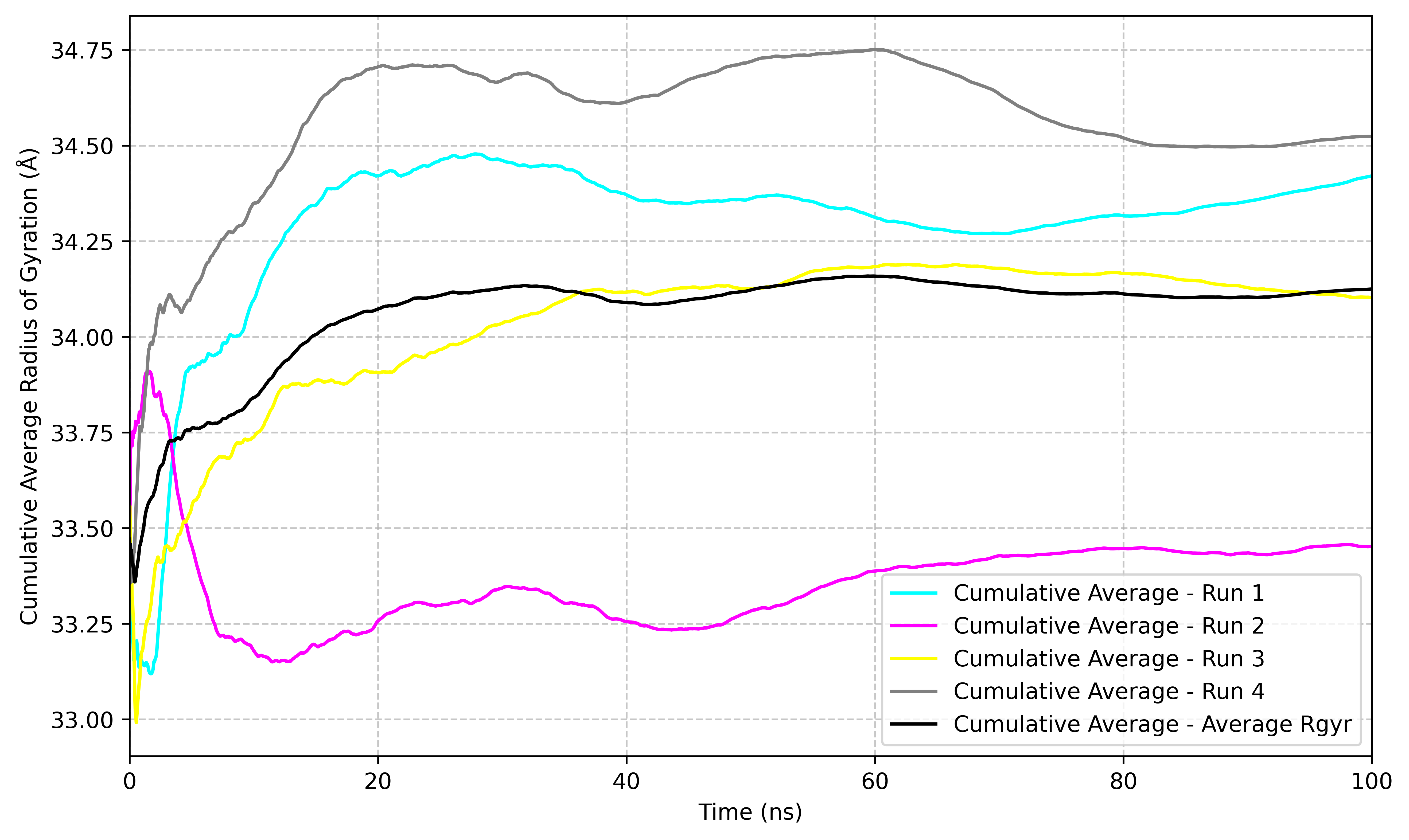

Supplement: Supplementary file 1 [file biology-14-00639-s001.zip › Supplemental Figures/Figure S9. Radius of gyration cumulative average plot for the reference ligand.png]
